# Proteomic analysis reveals dexamethasone rescues matrix breakdown but not anabolic dysregulation in a cartilage injury model

**DOI:** 10.1016/j.ocarto.2020.100099

**Published:** 2020-09-05

**Authors:** Rebecca Mae Black, Yang Wang, André Struglics, Pilar Lorenzo, Viveka Tillgren, Martin Rydén, Alan J. Grodzinsky, Patrik Önnerfjord

**Affiliations:** a Department of Biological Engineering, Massachusetts Institute of Technology, Cambridge, MA, USA; b Orthopaedics, Department of Clinical Sciences Lund, Faculty of Medicine, Lund University, Lund, Sweden; c Rheumatology and Molecular Skeletal Biology, Department of Clinical Sciences Lund, Faculty of Medicine, Lund University, Lund, Sweden; d Department of Mechanical Engineering, Massachusetts Institute of Technology, Cambridge, MA, USA; e Department of Electrical Engineering and Computer Science, Massachusetts Institute of Technology, Cambridge, MA, USA

**Keywords:** Post-traumatic osteoarthritis, Mass spectrometry, Cartilage matrix, Cytokines, Proteomics, Dexamethasone

## Abstract

**Objectives:**

In this exploratory study, we used discovery proteomics to follow the release of proteins from bovine knee articular cartilage in response to mechanical injury and cytokine treatment. We also studied the effect of the glucocorticoid dexamethasone (Dex) on these responses.

**Design:**

Bovine cartilage explants were treated with either cytokines alone (10 ng/ml TNFα, 20 ng/ml IL-6, 100 ng/ml sIL-6R), a single compressive mechanical injury, cytokines and injury, or no treatment, and cultured in serum-free DMEM supplemented with 1% ITS for 22 days. All samples were incubated with or without addition of 100 nM Dex. Mass spectrometry and Western blot analyses were performed on medium samples for the identification and quantification of released proteins.

**Results:**

We identified 500 unique proteins present in all three biological replicates. Many proteins involved in the catabolic response of cartilage degradation had increased release after inflammatory stress. Dex rescued many of these catabolic effects. The release of some proteins involved in anabolic and chondroprotective processes was inconsistent, indicating differential effects on processes that may protect cartilage from injury. Dex restored only a small fraction of these to the control state, while others had their effects exacerbated by Dex exposure.

**Conclusions:**

We identified proteins that were released upon cytokine treatment which could be potential biomarkers of the inflammatory contribution to cartilage degradation. We also demonstrated the imperfect rescue of Dex on the effects of cartilage degradation, with many catabolic factors being reduced, while other anabolic or chondroprotective processes were not.

## 1 Introduction

While glucocorticoids have been used for over 50 years to treat osteoarthritis (OA) pain, the prescription of glucocorticoids remains controversial because of potentially harmful side effects to multiple joint tissues, especially cartilage. One glucocorticoid, dexamethasone (Dex) has been demonstrated to rescue the loss of aggrecan and collagen constituents as well as chondrocyte viability in human and bovine cartilage explant models of inflammatory tissue injury and post-traumatic OA (PTOA) [1,2], suggesting the possibility of Dex as a disease-modifying drug. However, literature covering the effects of Dex on cartilage reveals conflicting results on the drug's safety profile [3]. Importantly, the anti-catabolic versus pro-catabolic effects of Dex on the cartilage extracellular matrisome, as well as the fate of intracellular proteins in the presence of tissue injury, remain unexplored. The goals of the present study are to utilize a discovery proteomics approach to quantify the loss of extracellular and intracellular proteins from cartilage explants subjected to inflammatory cytokine challenge and impact mechanical injury, and to determine whether Dex protects against or exacerbates the response.

Li et al. [2] demonstrated in an interleukin-1 (IL-1) challenge of full-thickness near-normal human cartilage explants that culture with 100 nM Dex continuously over a 17-day treatment rescued sulfated glycosaminoglycan (sGAG) loss and maintained more viable cells, relevant to potential PTOA prevention. Dex also rescued the cytokine-induced decrease in sGAG synthesis in this disease model, though not reaching control levels. These results showed beneficial effects of Dex on cartilage metabolism and extracellular matrix (ECM) synthesis in a model of early PTOA. Using human tissue in a tumor necrosis factor alpha (TNFα), interleukin-6 (IL-6), and single compressive injury challenge of normal human knee explants, Lu et al. [1] found that continuous Dex treatment rescued sGAG loss. In a similar cytokine model, a targeted proteomics approach identified increased levels of ECM components released to the media, including aggrecan, cartilage oligomeric matrix protein (COMP), and collagen III, compared to untreated explants [4].

At the same time, deleterious effects of Dex on cartilage tissue have been reported. Several studies using isolated human chondrocytes showed that even low doses of Dex can cause cell death and reduce cell proliferation, suggesting potential cytotoxic and catabolic side effects. However, the observed effects of Dex depends greatly on dose, model, duration of treatment and context (*e.g.*, isolated cells *versus* intact cartilage). Thus, study conclusions often differ greatly, even when studying intact cartilage explants, complicating the discussion of safety and efficacy of Dex [3].

These disparate results on cartilage tissue response to Dex have typically focused on select few matrix macromolecules without the benefit of a more encompassing view that could be provided by a systems-level analysis of changes to cartilage. Thus, the specific objectives of the present study are to (1) use a global discovery proteomics approach to quantify the effects of inflammation and mechanical impact injury on cartilage explants, and (2) study the effects of Dex in the presence and absence of inflammatory and injurious mechanical challenges. We believe this approach can lead to an increased understanding of the potential benefits of a glucocorticoid such as Dex, as well as the identification of biomarkers of cartilage degradation in the presence and absence of such treatments.

## 2 Material and methods

### 2.1 Explant harvest and culture

**Fig. 1:**
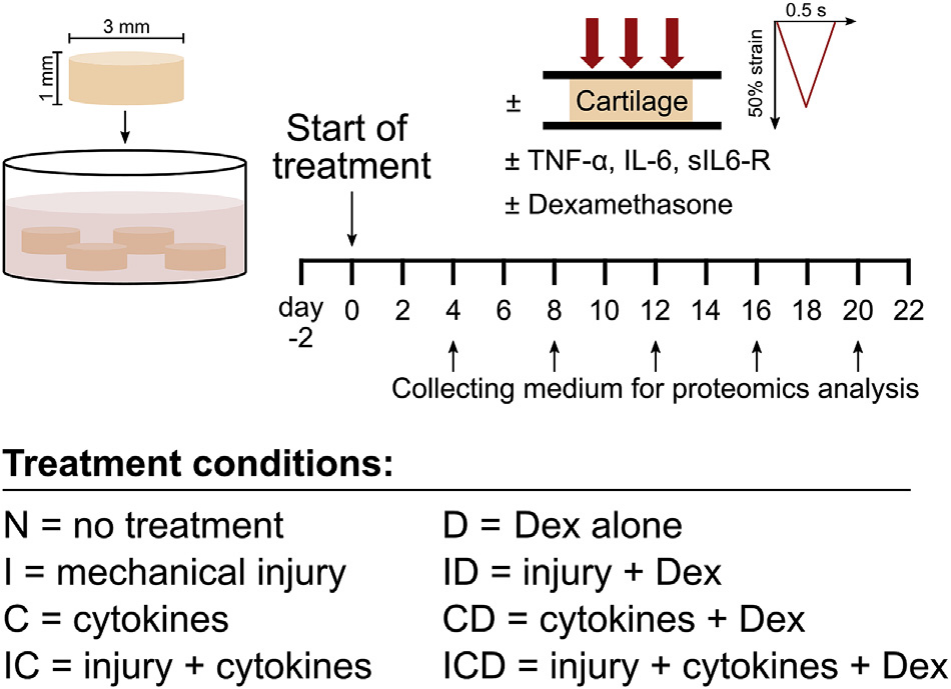
**Experimental setup**. Bovine articular cartilage explants (3 mm × 1 mm cylinders including the superficial zone, 4 per well) were cultured for three weeks according to the following treatment conditions: (N) untreated controls; (I) a single applied mechanical impact injury (50% final strain at a strain rate of 100%/s, followed by immediate release at the same rate); (C), addition of cytokines: TNFα, IL-6 and sIL-6R (10 ng/ml, 20 ng/ml, and 100 ng/ml, respectively) (IC), applied injury plus addition of cytokines; (D), untreated control + Dex (100 nM); (ID), applied injury + Dex; (CD), cytokines + Dex; or (ICD), applied injury + cytokines + Dex. Culture medium was changed every two days, and three biological replicates (animals) were used for all treatment conditions.

Cartilage disks (3 mm × 1 mm thick including the intact superficial zone) were harvested from the femoropatellar grooves of 1-2-week-old bovines (Research ‘87, Boylston, MA) as described [2], Fig. 1. One knee joint from each of three different animals were used. After harvesting, explant disks were pre-equilibrated for two days in serum-free medium (low-glucose phenol-red free Dulbecco's Modified Eagle's Medium (DMEM), ThermoFisher Scientific) supplemented with 10 mM HEPES buffer (Gibco), 2 mM l-Glutamine (Gibco), 0.1 mM nonessential amino acids (Sigma), 0.4 mM proline (Sigma), 20 μg/ml ascorbic acid (Sigma), 100 units/ml penicillin G, 100 μg/ml streptomycin, and 0.25 μg/ml amphotericin B (Sigma), and 1% insulin-transferrin-selenium (10 μg/ml, 5.5 μg/ml, and 5 ng/ml, respectively; Sigma).

### 2.2 Explant treatments

After pre-equilibration, samples were treated for 22 days as in Fig. 1: no treatment (N), mechanical injury at day 0 (a single unconfined compression at 50% final strain, 100%/s strain rate, followed by immediate release at the same rate [5]; treatment I); cytokines (10 ng/ml recombinant human TNFα, 20 ng/ml recombinant human IL-6 and 100 ng/ml soluble IL-6 receptor (sIL-6R) (R&D Systems); treatment C); injury + cytokines (treatment IC), with all four treatment groups additionally receiving 100 nM Dex (treatments D, ID, CD, and ICD, respectively). Medium changes were carried out every two days and collected medium was stored at −80 °C until analysis.

### 2.3 Mass spectrometry preparation and identification

Culture medium (50 μL) from days 4, 8, 12, 16, and 20 (Fig. 1) was prepared for mass spectrometry (MS) analysis as described (**Supplemental Methods**) [4]. Discovery MS was performed on medium samples using a quadrupole Orbitrap benchtop mass spectrometer (Q-Exactive, Thermo Scientific). Identification was performed using the UniProt Bos Taurus database (UP_000009136, 2017–10) with Proteome Discoverer 2.2 (Thermo Scientific). The protein false discovery rate (FDR) was 0.01. Label-free protein abundance quantification was obtained by summing peak area intensities from multiple unique peptides for each protein.

### 2.4 Data analysis

Proteins identified in all three animals were filtered, keeping only proteins identified and quantified in at least 5% of samples. Assuming that missing values were due to low abundance, missing values were imputed using quantities equal to half the lowest identified abundance for each protein. Abundance data were log_2_-transformed and principle component analysis (PCA) was performed on treatments N, I, C, and IC using the “prcomp” function in the R-package factoextra, with ‘center’ and ‘scale’ set to true to z-score values. Pairwise comparisons between treatments were performed on the summed peptide abundance over all five timepoints. Statistical analysis was performed using the R package limma, comparing log_2_-fold changes within each animal [6]. *P*-values were obtained using empirical Bayes statistics. Adjusted *p*-values were calculated using the Benjamini-Hochberg method with a *q*-value of 0.05.

### 2.5 Grouping of shared protein responses

Proteins were classified into three groups based on their response to treatments C and IC compared to control. The grouping criteria were: (I) decreased release in treatments C and/or IC, (II) increased release by treatment C alone, or (III) increased release in IC with or without increased release in C. Proteins were selected if they had a differential effect from the control (*p* < 0.05) and were present in at least three timepoints for one treatment condition across all three animals. Grouping criteria were based on patterns identified with hierarchical clustering of proteins with differential release from control, and validated against the raw, non-imputed data by two operators (AS, PÖ).

### 2.6 Enrichment analysis

Because of limitations in the annotation of the bovine proteome, we translated bovine accession numbers into human equivalents using blastp protein searches [7]. Enrichment analysis was performed by searching the Gene Ontology (GO) and STRING databases for biological process, molecular function, and Kyoto Encyclopedia of Genes and Genomes (KEGG) pathways [[8], [9], [10]]. Protein subcellular localization was determined via UniProt annotation [11]. *P*-values for intra- and extracellular groupings were determined through bootstrapping with all differentially expressed proteins as background and 10,000 repeats to generate estimated distributions [12].

### 2.7 Biochemical and Western blot analysis of aggrecan and cartilage oligomeric matrix protein (COMP) fragments

The amount of sGAG released to the culture medium over the 22-day culture was determined using the 1,9-dimethylmethylene blue assay (DMMB) [13]. The same samples were deglycosylated with chondroitinase ABC, keratanase and keratanase II as described (**Supplemental Methods** [14]), with the exception that keratanase II incubation was done for 3 h with 0.01 mU/μg sGAG. Deglycosylated samples were precipitated and electrophoresed as described [15], separated by sodium dodecyl sulfate-polyacrylamide gel electrophoresis (SDS-PAGE) and transferred to polyvinylidene difluoride (PVDF) membranes. Immunoreactions were performed using *anti*-ARGS aggrecan neoepitope antibodies [16] or *anti*-G3 aggrecan polyclonal antibodies (all tested for specificity [17]) and immunobands were visualized with secondary horse anti-mouse peroxidase-conjugated antibodies using enhanced chemiluminescence (ECL).

The breakdown of COMP was analyzed as described (**Supplemental Methods** [18,19]). Twenty microliters of culture media were taken every second day from a separate set of bovine explant cultures (treatments N and IC, in the presence and absence of the combination of 100 nM Dex and continuous dynamic compression (10% strain amplitude, described previously [5])). Samples were separated by SDS-PAGE, transferred to a nylon membranes and incubated with rabbit anti-bovine COMP polyclonal antiserum [19]. Immunobands were visualized with secondary peroxidase-conjugated antibodies using ECL. Purified bovine COMP [19] was used as a reference.

## 3 Results

**Fig. 2:**
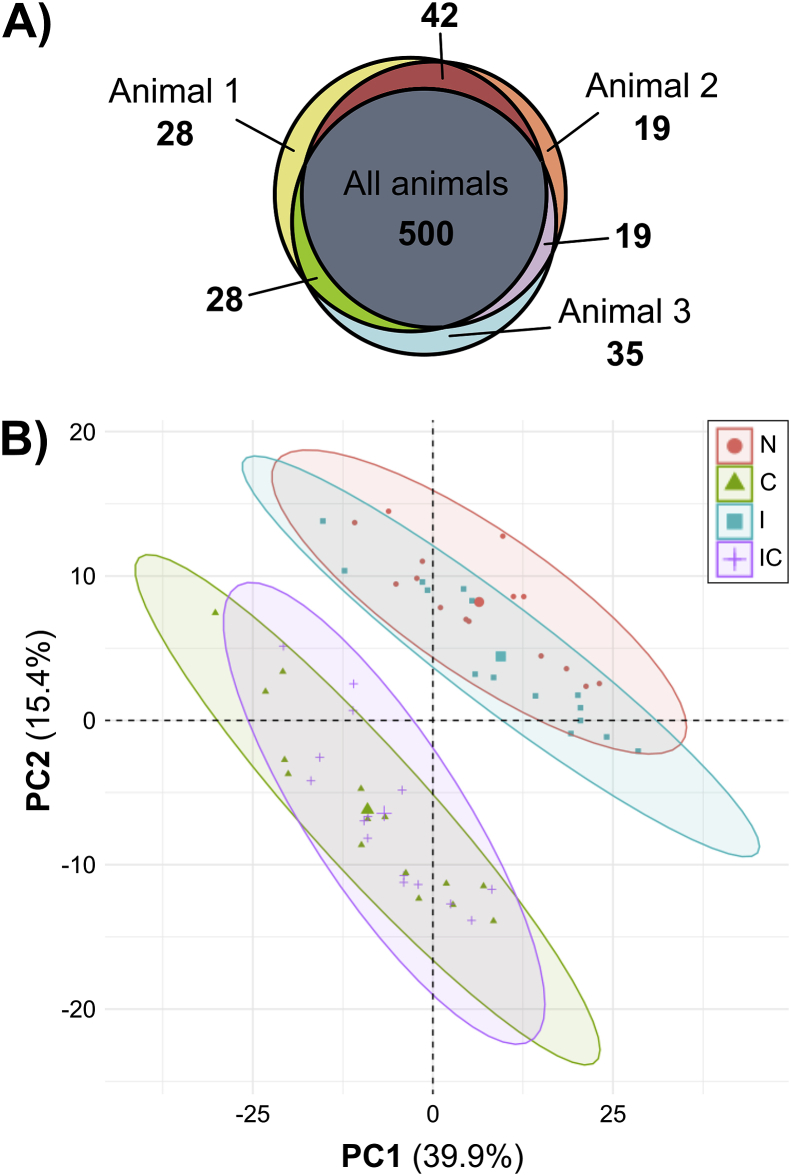
**Proteomic overview of the three biological replicates**. (A) A Venn diagram reveals a large overlap of MS-identified proteins between biological replicates. 500 proteins were found in medium samples from all three animals, and few proteins were found in only one or two animals. (B) Principle component analysis (PCA) was performed using abundance values for 456 filtered proteins obtained from MS analysis of cartilage explant medium samples taken on day 4, 8, 12, 16, and 20 of culture from the treatment groups control (N), injury (I), cytokines (C), and injury + cytokines (IC). The data clearly separate into two clusters, one with treatments N and I, and the second with treatments C and IC. Percentages on axes represent percent variance explained by that principal component. The large symbol within each cluster represents the cluster centroid.

MS analysis identified 671 proteins; 500 proteins were found in all three animals (Fig. 2A). The raw data are available via ProteomeXchange with identifier PXD020756 [20]. After filtering as described in Methods, the data set was reduced to 456 proteins (Supplemental File 1). An example of the complete data for an individual protein (matrix metalloproteinase-1, MMP-1) is depicted in Supplemental Fig. S1. PCA clustering on log_2_-transformed abundance data (Fig. 2B, all eight treatment groups in Supplemental Fig. S2) revealed cytokine treatment as the major determinant of medium composition: one cluster is seen for control and injury (N, I) and another cluster for samples treated by cytokines with and without injury (C, IC). Mechanical injury only (treatment I) had a marginal effect on this cytokine-dependent clustering. Therefore, treatment I was excluded from the grouping criteria because of the low number of proteins significantly affected by that treatment and its similarity to control.

### 3.1 Comparison between explant treatments

**Fig. 3:**
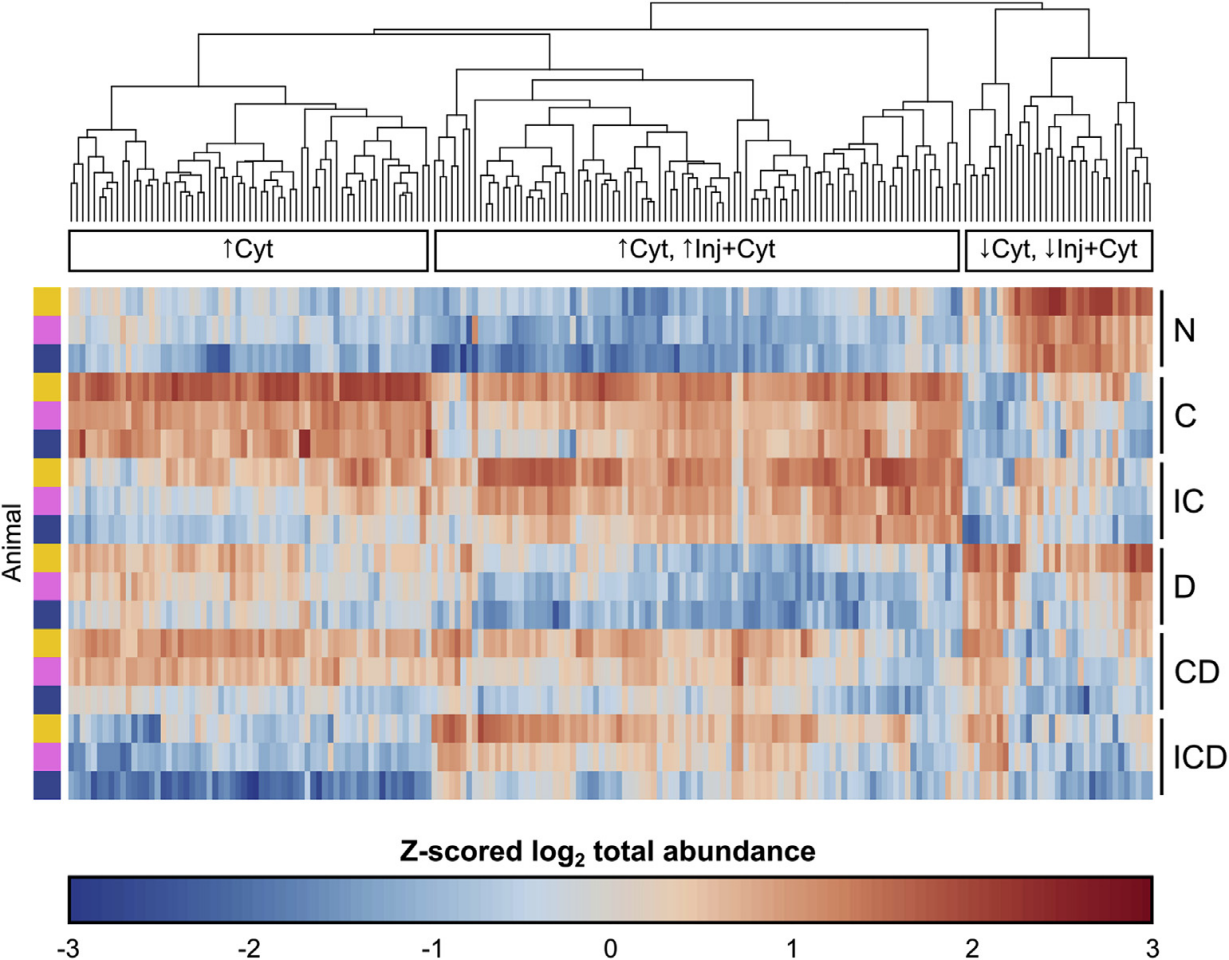
**Heatmap of proteins significantly affected by disease treatment**. Treatment effects were evaluated by pairwise comparisons of MS abundance data (log_2_ summed ratios with imputation of missing values, FDR = 0.05) of different disease treatments within each animal replicate. Proteins were selected that had a differential effect of C or IC treatments and that were present in at least three time points across at least one consistent treatment condition between all three biological replicates (to avoid biases from imputing missing values), resulting in 188 selected proteins. The raw abundance value for each filtered protein was summed over all timepoints and log_2_-transformed. For visualization, the log_2_-transformed values were normalized via z-scoring across all treatment conditions, excluding injury alone and injury with Dex: control (N), cytokine (C), injury + cytokines (IC), Dex (D), cytokines + Dex (CD), and injury + cytokines + Dex (ICD). Proteins are plotted on the horizontal axis, and ordered based on their hierarchical clustering (Euclidian distance) across all six selected treatment conditions. Each individual replicate is plotted on the vertical axis, ordered by treatment condition and then by animal. The clustering reveals three major patterns of protein release: increased release by cytokines alone (↑Cyt), an increase by both cytokines and injury + cytokines (↑Cyt, ↑Inj + Cyt), and decreased release by cytokines and injury + cytokines (↓Cyt, ↓Inj + Cyt). These three patterns were used to designate the grouping categories in Table 1.

Pairwise comparison between treatments showed a major effect of treatments C and IC: 175 proteins (149 up, 26 down) and 115 proteins (94 up, 21 down) respectively, were observed to have a difference from control (Supplemental File 2). 195 proteins in total had a differential effect of treatments C and IC versus control, and 88 proteins had a differential effect of Dex on control, cytokines, or injury + cytokines (FDR = 0.05). After filtering for proteins present in at least one consistent treatment condition across all three animals, there were a resulting 188 proteins differentially released by treatments C or IC (Fig. 3; results for all eight treatment groups in Supplemental Fig. S3).

### 3.2 Protein selection into similar signal-response groups

**Fig. 4:**
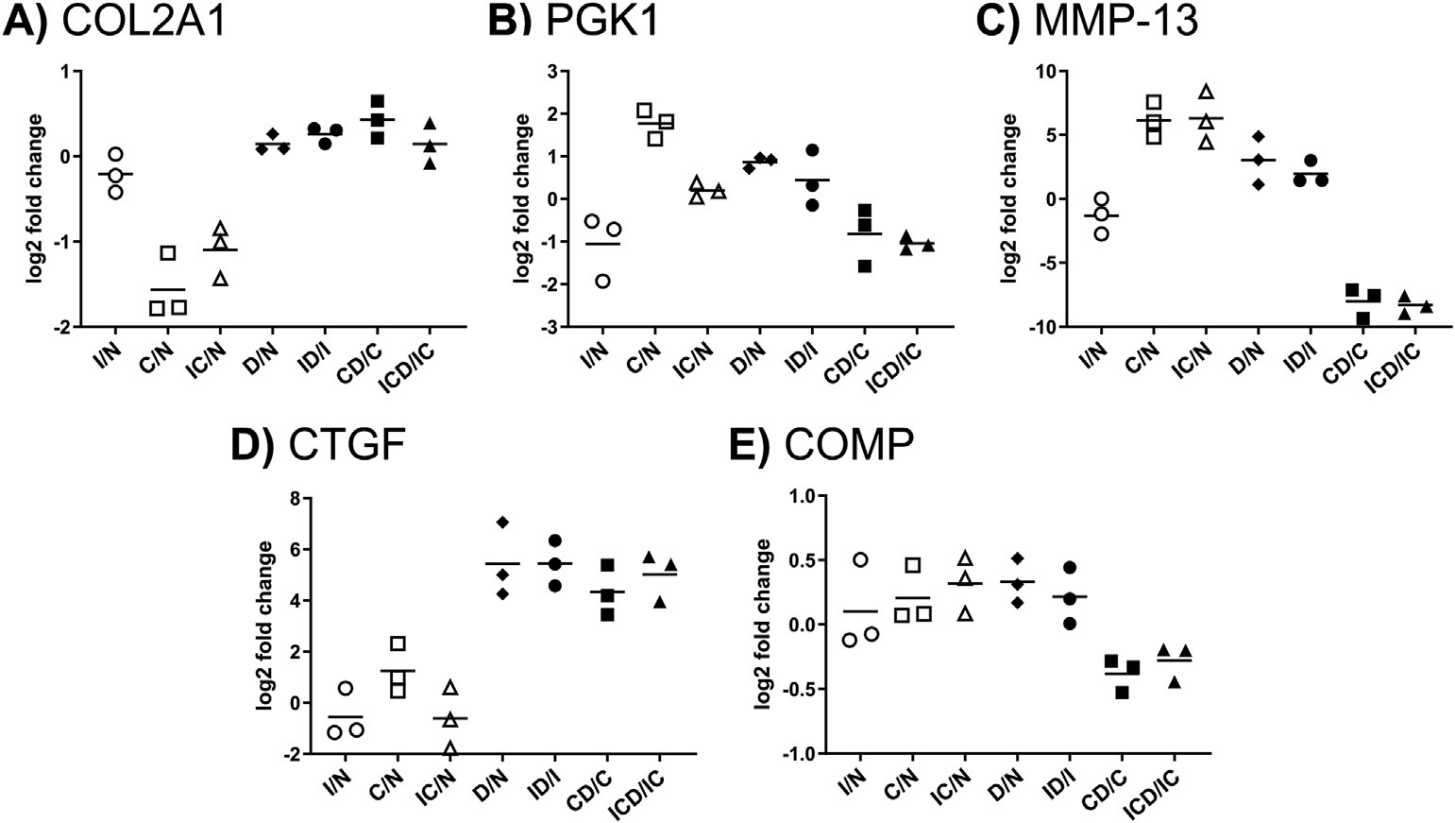
**Representative proteins for each response profile**. The simplified graphical representation for each protein shows the ratio for each treatment comparisons: injury (I), cytokines (C), and injury + cytokines (IC) versus control (N), and treatments with Dex versus their non-Dex controls: D *vs*. N, ID *vs*. I, CD *vs*. C, and ICD *vs*. IC. The mean fold change values from three replicates are indicated with horizontal lines. Representative proteins for the different profile categories (Table 1): (A) Group I, collagen type II (COL2A1); (B) Group II, phosphoglycerate kinase (PGK1); (C) Group III, collagenase-3 (MMP-13). Also shown are (D) connective tissue growth factor (CTGF), a representative of proteins with a consistent effect of Dex after addition to any treatment, and (E) cartilage oligomeric matrix protein (COMP), which undergoes no statistically significant change in release with any treatment.

The 188 filtered proteins were classified into three response groups (Table 1, see Supplemental Table S1 for full protein names), illustrating treatment effects as well as the effects of Dex intervention. Compared to control, 156 proteins (83% of the 188 selected proteins) had increased release in conditions C and IC, while 32 proteins (17%) were decreased. We selected one representative protein from each response group with a clear representation of that group's trend to illustrate treatment effects in the presence and absence of Dex: type II collagen (COL2A1), phosphoglycerate kinase 1 (PGK1), and collagenase-3 (MMP-13) (Fig. 4A–C). We further categorized the most highly enriched GO annotations for biological process and molecular function (Supplemental Table S2). Groups I and III had a high proportion of extracellular proteins (67% and 51% (*p* < 0.0001), respectively, versus 42% for the entire population of 188 proteins). Group II was enriched (*p* < 0.0001) for intracellular proteins (78% versus 47% in the entire population), while consisting of only 4% extracellular proteins.

**Table 1:** 188 proteins grouped by response profile and proteins with consistent Dex effect. 188 selected proteins were categorized into subgroups (I-III) based on their shared release profiles under different treatment conditions. Those proteins with the same Dex effect in every treatment condition (D, CD, or ICD) are also listed. Proteins increased by Dex treatment are bolded, those decreased by Dex have no formatting, and proteins with no change with Dex are italicized.

I (n = 32)	II (n = 64)		III (n = 92)		DEX (n = 6)	
Inhibition by C and/or IC	Increase by C alone		Increase by IC alone or C and IC		Dex effect in every condition	
**ELN**	**AGT**	*PCBP1*	**DKK3**	*CKAP4*	**AGT**	**Increased with Dex**
**IGFBP4**	*ACTA2*	*PCMT1*	**SAA1**	*COL6A1*	**CTGF**	Decreased with Dex
**IGFBP7**	*ACTB*	*PFN1*	**TNFRSF6B**	*COL6A2*	**SERPINA1**	*No change with Dex*
**LOX**	*ACTN4*	*PGAM1*	ANXA8	*COL6A2_2*	**SPOCK1**	
**SERPINA1**	*ARCN1*	*PGD*	BMP1	*ECM1*	NUCB1	
**SPOCK1**	*ARHGDIA*	*PGK1*	C1S	*EEF1A1*	RPL36A	
**SPON1**	*BLVRB*	*PGM1*	CATHL1	*EEF1D*		
COL9A2	*CALM1*	*PHGDH*	CCL5	*EFNA1*		
ECRG4	*CAPN2*	*PKM2*	CDA	*FN1*		
IGF2	*CFL1*	*PPIA*	CHI3L1	*H2AFZ*		
*AEBP1*	*CNPY4*	*PRDX2*	CSF1	*HIST1H1D*		
*CDON*	*COPE*	*PRDX5*	EEF1G	*HIST1H2AC*		
*CGREF1*	*CTSB*	*PRDX6*	FBN2	*HIST1H2AJ*		
*CHRDL2*	*DBI*	*PSMB5*	GDF6	*HIST1H4D*		
*CLSTN1*	*DSTN*	*PSMB6*	HAPLN3	*HIST2H2BF*		
*CNMD*	*EFEMP1*	*RAN*	HP	*HIST2H3PS2*		
*COL2A1*	*FLNB*	*RPL10A*	IGFBP5	*HMGB1*		
*COL9A3*	*FSCN1*	*RPL12*	INHBA	*HMGN3*		
*CTHRC1*	*GAPDH*	*RPLP0*	LGALS1	*HNRNPA3*		
*EDIL3*	*GSTM2*	*RPLP2*	LTBP1	*HSPE1*		
*EGFR*	*GSTP1*	*RPS21*	MMP1	*IGFBP3*		
*EIF5B*	*HBA1*	*RPSA*	MMP13	*LCN2*		
*GOLM1*	*HNRNPA1*	*RRBP1*	MMP3	*LGALS3*		
*GREM1*	*HNRNPA2B1*	*ST13*	MMP9	*LMNA*		
*LTBP3*	*HNRNPD*	*TARS*	OAT	*LMNB1*		
*MIA*	*HSP90AA1*	*TPM3*	OLFML2B	*LMNB2*		
*NUDC*	*HSP90AB1*	*TPT1*	PLEC	*LTBP2*		
*PROS1*	*HSP90B1*	*TUBA1D*	S100A2	*LUM*		
*PSMA6*	*HSPA8*	*TXNDC5*	SEMA3C	*M-SAA3.2*		
*SCG5*	*LASP1*	*Unknown*^a^	SERPING1	*MT2*		
*SULF2*	*MAP4*	*VIM*	TGFB2	*MYL6*		
*SUSD5*	*NCL*	*YBX1*	THBS2	*OLFML3*		
			VASN	*ORM1*		
			VCAM1	*PRELP*		
			*ADAMTSL4*	*RBMX*		
			*ALYREF*	*S100A4*		
			*ANXA2*	*SAA3*		
			*APOD*	*SDC4*		
			*ATP5A1*	*SERPINC1*		
			*C1R*	*SERPINE1*		
			*C3*	*SERPINH1*		
			*CAPG*	*SOD2*		
			*CCDC80*	*TMA7*		
			*CD14*	*TNC*		
			*CD44*	*TNFRSF11B*		
			*CFB*	*TTR*		

a Accession #G5E6G2.

### 3.3 Protein network analysis

**Fig. 5:**
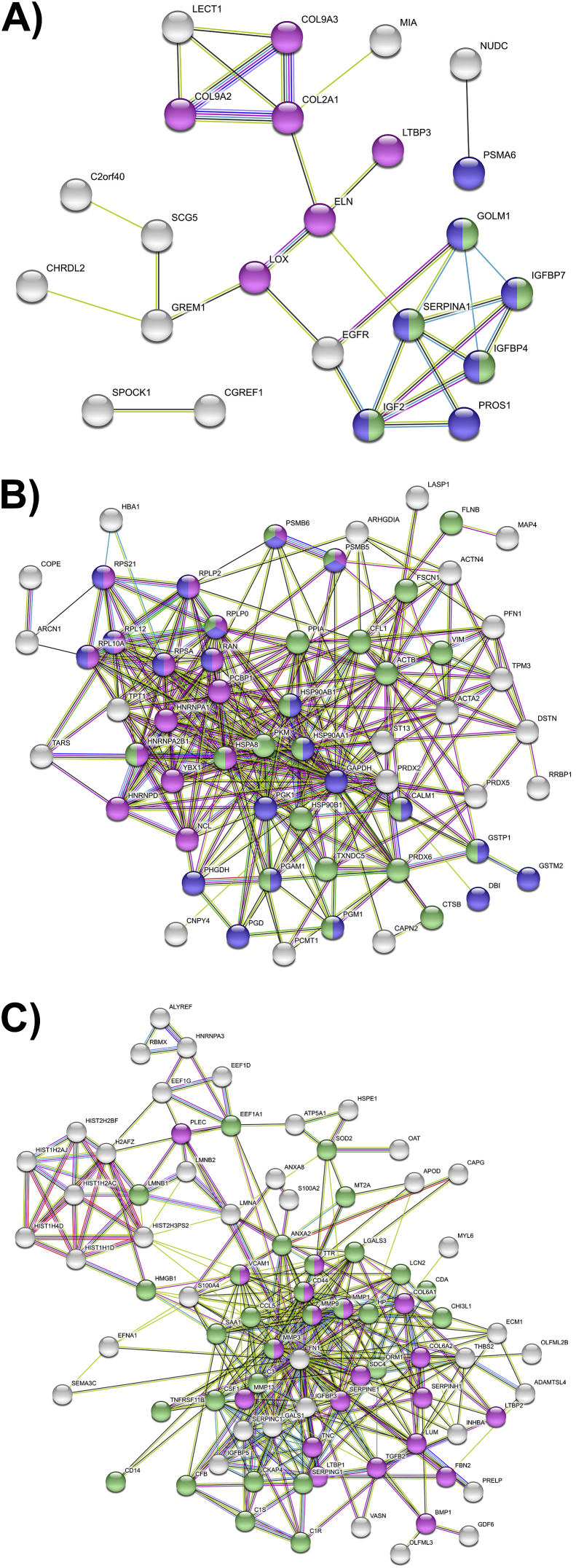
**STRING network analysis plots**. STRING network maps of proteins in each of the three groups of shared release profiles, colored based on KEGG pathway enrichment. Unconnected nodes were removed from network maps. (A) Group I: proteins with decreased release after C or IC treatment. Magenta: ECM and ECM-modifying proteins; blue: metabolism; green: IGF transport and uptake. (B) Group II: proteins with increased release after C treatment only. Magenta: RNA-metabolizing proteins; blue: metabolism; green: immune system. (C) Group III: proteins with increased release after C and IC treatment. Magenta: ECM organization; green: immune system.

STRING network analysis revealed two small network interaction clusters for proteins in Group I (Fig. 5A): ECM-related proteins including collagens II and IX, and a cluster enriched for protein metabolism and IGF transport and uptake. The network map for Group II (Fig. 5B) consisted of one large, highly interconnected cluster enriched for immune and RNA-metabolizing proteins, as well as a diverse subset of proteins involved in metabolic processes, including glycolysis, redox homeostasis, endoplasmic reticulum function, and protein synthesis. The pathways enriched in the largest cluster of Group III were ECM organization and the immune system, with a distinct cluster of histone proteins (Fig. 5C).

### 3.4 Dexamethasone treatment

The effects of Dex on the release of the 188 selected proteins to the medium is shown in Table 1. Compared to treatments without Dex, 34 proteins (18%) had significantly decreased signal in the presence of Dex, while 11 proteins (5.4%) had increased signal. In the largest protein group (III), 31 of the 92 proteins (34%) showed reduced release following Dex treatment. A small number of proteins experienced an effect in every condition with Dex compared to that condition without Dex (Table 1), exemplified by connective tissue growth factor (CTGF) in Fig. 4D.

### 3.5 Release of aggrecan and COMP fragments

**Fig. 6:**
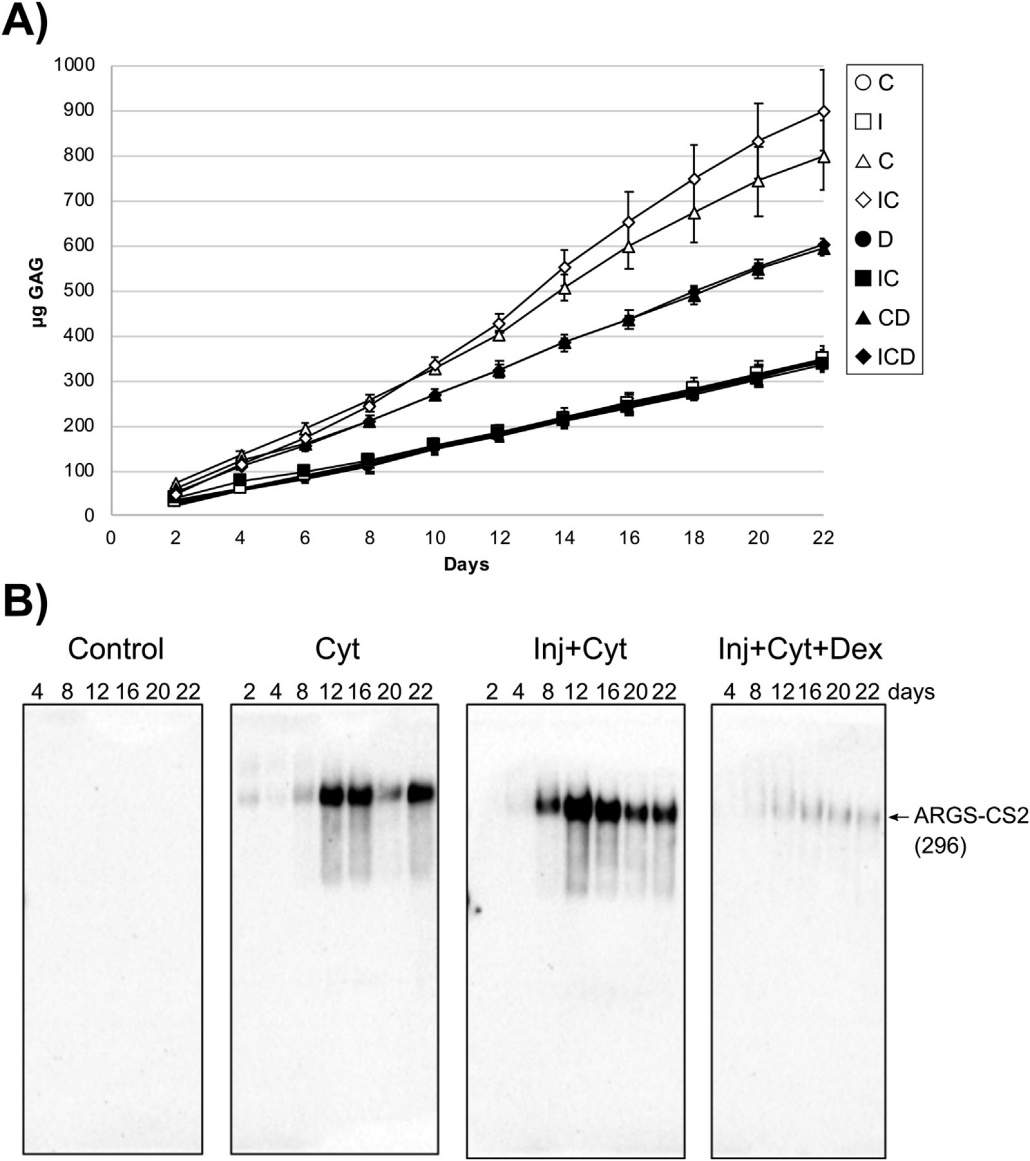
**Time dependent release of aggrecan constituents into explant culture media**. (A) Cartilage explants (n = 3) were cultured from 2 up to 22 days. The amounts of sGAG (mean ± SD) released at each day of culture was measured by the DMMB assay for all treatment groups: control (N), injury alone (I), cytokine (C), injury + cytokines (IC), Dex (D), injury + Dex (ID), cytokines + Dex (CD), and injury + cytokines + Dex (ICD). The release of sGAG was elevated with cytokine treatment; the addition of Dex reduced this release. (B) Medium samples were deglycosylated and run (44–100 μL medium/lane) on 3–8% Tris-acetate SDS-gels and applied for Western blot using ARGS-aggrecan N-terminal neoepitope antibodies. Bovine protein fragments (previously described [21]) and their molecular weight in kDa are shown at the right. CS2 = chondroitin sulfate region 2. Cyt = cytokine treatment, Inj = applied injury, Dex = Dex treatment.

Cytokine and injury treatments had no effect on the release of aggrecan fragments by proteomic analysis, but were evaluated in parallel by the DMMB assay for sGAG release (Fig. 6A). Treatments C and IC caused a 2- to 3-fold increase in sGAG release compared to controls; this release was significantly reduced by addition of Dex. Western blot analysis supported these findings indicated by the release of aggrecanase-generated ARGS-CS2 fragments (Fig. 6B), and by the release of smaller aggrecanase generated G3-CS2 fragments [17] upon treatment with cytokines and injury + cytokines (Supplemental Fig. S4). The release of these aggrecan fragments was rescued by treatment with Dex.

While our MS analyses of COMP release in the present study indicated no clear treatment effects (Fig. 4E), Western blots indicated a dramatic increase in COMP degradation manifested by the release of smaller COMP fragments after addition of injury + cytokines and a partial amelioration of COMP fragment release upon Dex treatment (Supplemental Fig. S5).

## 4 Discussion

This study incorporates a global discovery approach to characterize the effects of inflammation and mechanical injury on cartilage explants, including the role of therapeutic intervention with Dex. This glucocorticoid treatment has been used with intra-articular injection for treatment of arthritic or post-surgery knee pain [[22], [23]] with sometimes disappointing results [24], due in part to rapid clearance from the joint space before penetration into cartilage [[25], [26], [27]]. In some clinical trials with glucocorticoids, very high, frequent doses were used, leading to adverse effects such as a reported decrease in cartilage volume without any reduction in knee pain [28]. However, *ex vivo* explant studies have shown that sustained low doses of Dex can rescue sGAG loss, reduce proteolytic enzyme synthesis, and maintain chondrocyte viability under inflammatory cytokine challenge [2].

The present study is based on label-free protein quantification allowing a semi-quantitative estimation of relative protein abundances. Cytokine treatment, in particular, exhibits a large effect on proteins released into the explant culture media, while treating with mechanical injury alone has a small effect, as demonstrated by the overlap of the two treatments in PCA space (Fig. 2B).

### 4.1 Catabolic processes are activated under arthritic stress

After an acute joint injury, the progression of PTOA is initiated by an early response characterized by the production of inflammatory factors and proteases, including MMPs, in reaction to inflammatory stress and chondrocyte death [29]. The transport into cartilage of inflammatory factors released from the synovium is thought to be further enhanced by microdamage to the cartilage surface caused by mechanical impact [30]. Together, these changes lead to progressive degeneration of the ECM which can cascade into irreversible matrix loss and PTOA [31].

In our model, we confirmed the PTOA-like effects of IL-6 and TNFα on bovine cartilage explants by examining the release of sGAGs over the three-week course of the experiment (Fig. 6A). We found an increased cumulative release of sGAG into the culture media upon cytokine treatment, consistent with previously published data from a similar experiment [1]. The sGAG release is associated mainly with proteolysis of aggrecan, as confirmed by release of ARGS-CS2 and G3-CS2 fragments (Fig. 6B and S2).

### 4.2 Groups II and III demonstrate cartilage catabolic response to inflammatory and mechanical stress

Group III, proteins with increased release after exposure to inflammatory cytokines with or without applied injury, best represents the catabolic response of cartilage to PTOA stress. This group is the largest and represented by MMP-13 (Fig. 4C), an active protease in the breakdown of a range of cartilage proteins in OA [32]. Protein categories in Group III include proteases and protease inhibitors (ex. MMP-1, -3, -9, -13, plasma protease C1 inhibitor, plasminogen activator inhibitor 1, and serpin H1) signaling (ex. C–C motif chemokine 5, ephrin-A1, inhibin, and transforming growth factor beta-2 (TGFβ-2)) and ECM (ex. collagen VI, fibrillin-2, fibronectin, tenascin). These results suggest that Group III proteins are those actively released from the cartilage after the induction of PTOA: the degradation products of cleaved ECM components and the proteases, protease inhibitors, and other signaling factors associated with catabolic responses.

Inflammatory stress also disrupts intracellular processes and leads directly to chondrocyte death [2]. The dysregulation of chondrocyte homeostasis under disease stress can be observed within Group II proteins, whose release was increased by exposure to inflammatory cytokines alone and consist of majority intracellular proteins. The protein representing this group is PGK1, (Fig. 4B), a major enzyme in the glycolytic pathway. It is notable that many of the proteins in this group are involved in cellular metabolism (ex. glyceraldehyde-3-phosphate dehydrogenase, l-lactate dehydrogenase, PGK1, phosphoglycerate mutase 1), endoplasmic reticulum processing (ex. 78 kDa glucose-regulated protein, protein disulfide-isomerase A6, calreticulin), or ribosomes (ex. ribosomal proteins L10A, L12, P0, and P2). The biological reason for the increased release of these proteins may be due to transcriptional or translational changes in response to cytokine treatment, or a result of cell death causing these highly abundant intracellular proteins to be released from necrotic cells.

### 4.3 Dexamethasone rescues some catabolic processes

The increased release of one third of the proteins in Group III was partially or completely rescued by Dex, including all four MMPs in this group. This finding agrees with literature supporting the observations that Dex inhibits the production of MMPs under arthritic stresses and prevents ECM breakdown as measured by sGAG loss and histological staining [3]. This effect of Dex was specific to the proteins in Group III from treatments CD or ICD: the proteins involved in ECM organization (including fibrillin, versican, five MMPs, TGF-β, and plasma protease C1 inhibitor) that Dex reduced from Group III were not affected by Dex alone, except a slight increase in MMP-13 release. However, Dex did not rescue the increased release of most of the proteins in Group II. Dex has been shown to protect against inflammatory cytokine-induced chondrocyte death [2], but many Group II proteins had high release at the earliest timepoint which was not prevented by Dex, suggesting that Dex may not prevent an early cellular response to inflammatory cytokines. This result may also be influenced by changes in protein synthesis. It has been hypothesized that Dex suppresses cartilage metabolism, but whether these effects are propagated through changes in protein synthesis has not yet been explored [3].

### 4.4 Some anabolic processes are inhibited by PTOA stress

As catabolic processes continue in diseased cartilage, chondrocytes begin to produce chondroprotective and anabolic factors that initiate reparative processes to reverse proteolytic and inflammatory damage [29]. Group I represents proteins exhibiting decreased release after C or IC treatment, many of which would contribute to protective and anabolic responses, represented by collagen II, the most abundant collagen in cartilage (Fig. 4A). Collagen IX has a similar pattern of release. It is likely that the reduced release of these proteins is due to decrease in their synthesis. With decreased release, the major effect on their release from cartilage would not be proteolysis or the increased permeability of degrading ECM. In a previous study using injury + cytokine-treated human knee cartilage, the release of the C-terminal pro-peptide of collagen II, a synthesis marker, was decreased [4]. The decrease in the synthesis of these proteins could be related to early suppression of fibrillogenesis of collagen II, which associates with collagens IX and XI to form thin collagen fibrils [33].

The release of lysyl oxidase (LOX), an enzyme that regulates fibrillogenesis and collagen synthesis [[34], [35]], was also inhibited by cytokine addition. Another member of Group I, chondromodulin (CNMD) maintains cartilage homeostasis by preventing hypertrophic differentiation and remodeling to bone [36]. The release of some proteins may be affected by the developmental state of the tissue used in our model: chordin-like-2 (CHRDL2) which inhibits chondrocyte mineralization, and the signaling protein collagen triple helix repeat containing-1 (CTHRC1), have been found to be increased in human osteoarthritic cartilage, chondrocytes, or synovial fluid in other studies [[37], [38], [39]]. However, CHRDL2 is also expressed in differentiating chondrocytes, and CTHRC1 is expressed in developing growth plate cartilage [40], so the effect on their release may be associated with the juvenile tissue used in this study and not a protective response.

Certain proteins identified in Group III also reveal an increase in some chondroprotective processes. For example, the glycoprotein osteoprotegerin (TNFRSF11B) can suppress osteoclastogenesis and bone resorption [[41], [42]]. Thrombospondin-2 (THBS2) is a chondrogenic growth factor, and chondrogenesis has been suggested as protective against OA progression [[43], [44]]. In contrast to collagen II and IX, collagen VI, a major component of the pericellular matrix [45], showed increased release after IC treatment. Overall, it appears that some chondroprotective and anabolic processes may be activated after cartilage experiences injury or inflammation, while others are inhibited, possibly reducing the ability of the cartilage to resist subsequent damage to the matrix.

### 4.5 The effect of Dex on anabolic and chondroprotective factors is inconsistent

Dex does not reverse the effect of inflammatory cytokines for many proteins in Group I. For example, the release of LOX and testican-1 (SPOCK1, a member of the SPARC family) was rescued up to or above control levels, but the release of SPARC was not affected by Dex, suggesting that the actions of Dex on cartilage under arthritic stress do not result in a perfect rescue. This is also reflected in the behavior of collagens II and IX, as Dex did not rescue or further decreased their release (which was attributed to a decrease in synthesis). Previous studies on the effects of Dex on collagen synthesis have often reported conflicting results [3].

### 4.6 Dex induces the release of proteins not present in any other condition

Highly relevant to the consideration of Dex as a PTOA disease-modifying drug are “side effects” of Dex: Dex causes a significant increase in the release of some proteins in all treatment conditions, such as CTGF (Fig. 4D). CTGF is directly activated by glucocorticoids in its upstream promoter region, and is involved in a complex signaling network involving TGF-β signaling and joint homeostasis [[46], [47]]. Dex is widely accepted to inhibit MMP production and/or activity [3], possibly in our model by stimulating the production of protease inhibitors such as SPOCK1, a pro-MMP2 inhibitor found in cartilage [48], and alpha-1-antiproteinase (SERPINA1), a broad protease inhibitor [49]. Our observation that Dex affects such proteins even without disease stress suggests that there may be some dysregulation in the maintenance of the cartilage matrix in healthy cartilage exposed to Dex during treatment. The effects and side effects of Dex will be affected by its concentration in the cartilage tissue: in this experiment, we used the low dose of 100 nM Dex, which has been shown to be effective in ameliorating sGAG loss in immature bovine tissue under inflammatory stress [1]. This dose is orders of magnitude lower than typical clinical injections, but novel targeted delivery methods can achieve a low, sustained dose in cartilage, removing the need for repeated high-dose injections of Dex to achieve clinical efficacy [[25], [26]].

### 4.7 Study limitations

Proteomics is a powerful tool to measure changes in global protein expression at a high level and identify changes to entire pathways, but has some limitations. This analysis utilized data from a small number (48) of single-peptide identifications, which are less reliable for quantitation than proteins with more peptide coverage, but kept for analysis because of their identification at a FDR of 0.01 and their presence in at least 5% of samples. An MS approach summing peptide abundances reflects total protein level and is less sensitive to small changes in cleavage events, such as with COMP and aggrecan. Our proteomics results do not reveal a clear effect on treatment with cytokines; in contrast, the western blots show clear proteolytic cleavage (COMP: Fig. 4E, Supplemental Fig. S5; aggrecan: Fig. 6B). Some limitations could be circumvented by using an enzyme other than trypsin, though every enzyme will experience limitations based on its cleavage sites. While adult human cartilage may show some differences in the process of cartilage breakdown, this bovine model has been well-established as a model of PTOA progression in a more accessible and repeatable way than human cartilage. Three animals is a low number of replicates for robust statistical analysis, though somewhat offset by the repeatability of this bovine model. Future repeats and validation will strengthen the identified trends. Some observations here may be due to the early developmental state of the cartilage. Ongoing studies using adult human cartilage explants will be compared to the bovine results, and investigate the effects of bone and synovial responses to inflammation and crosstalk with cartilage such as the development of OA-related osteoporosis or Dex side effects [50].

## 5 Conclusions

In this exploratory study, we used a discovery proteomics approach to follow the release of proteins in response to mechanical damage and cytokine treatment of bovine knee articular cartilage. We also investigated the effect of a low, continual dose of the glucocorticoid Dex. The majority of differentially expressed proteins were increased upon treatment but some proteins, including the fibrillar collagens II and IX, were reduced. A large number of the proteins with increased release after disease treatment had reduced release in the presence of Dex. These disease-induced proteins could be potential biomarkers of the inflammatory contribution to cartilage degradation and demonstrate the protective effect of Dex against matrix breakdown and protease release. Analyzing proteins released from the cartilage also allowed some insight into the dysregulation of anabolic processes after disease induction: the release of some anabolic factors was increased after cytokine exposure, suggesting attempts to protect against and repair catabolic effects, while many other anabolic factors had their release suppressed by cytokine exposure. Dex treatment had mixed effects on these changes, highlighting the need for further experiments to explore the effect of Dex on intracellular processes in tissue models of OA. The design of this study will allow for further exploration of regulation of protein release kinetics after disease or Dex treatment.

## Author contributions

All were involved in the conception and design of the study as well as interpretation of the data. YW, PL, VT, AS conducted the experimental work and data analysis. PÖ conducted the mass spectrometry work and data analysis. Bioinformatics analysis was mainly performed by MR and RMB. RMB and PÖ drafted the manuscript while all authors critically revised the manuscript and gave final approval of the article.

## Declaration of competing interest

The authors do not have any conflict of interest.

## Appendix ASupplementary data

Supplemental File 1 Quantitative data from 456 filtered proteins present in all three animals and with artefacts from culture removed. Accession number, assigned protein and gene name, unique peptides identified, Sequest HT peptide scores, and abundance data. Samples are identified by treatment condition, day of culture (4, 8, 12, 16, 20), and biological replicate (b1, 2, 3). ctrl = control, inj = mechanical injury, cyt = cytokine treatment, DEX = Dex treatment. Supplemental File 1

Supplemental File 2 TopTable statistical summary of pairwise treatment comparisons. Log_2_-fold changes are reported for each pairwise comparison, as well as average log_2_-transformed release across all conditions, F statistic, raw and adjusted *p*-values, and log-odds value (‘B’). Supplemental File 2

Multimedia component 3 Multimedia component 3

Supplemental Table S1 Table 1 with full protein names. Supplemental Table S1

Supplemental Table S2 Summary of response group Gene Ontology results for top four biological process and molecular function enrichments. FDR = false discovery rate. Supplemental Table S2

## References

[1] Lu Y.C.S., Evans C.H., Grodzinsky A.J. (2011). Effects of short-term glucocorticoid treatment on changes in cartilage matrix degradation and chondrocyte gene expression induced by mechanical injury and inflammatory cytokines. Arthritis Res. Ther..

[2] Li Y., Wang Y., Chubinskaya S., Schoeberl B., Florine E., Kopesky P. (2015). Effects of insulin-like growth factor-1 and dexamethasone on cytokine-challenged cartilage: relevance to post-traumatic osteoarthritis. Osteoarthritis Cartilage.

[3] Black R., Grodzinsky A.J. (2019). Dexamethasone: chondroprotective corticosteroid or catabolic killer?. Eur. Cell. Mater..

[4] Wang Y., Li Y., Khabut A., Chubinskaya S., Grodzinsky A.J., Önnerfjord P. (2017). Quantitative proteomics analysis of cartilage response to mechanical injury and cytokine treatment. Matrix Biol..

[5] Li Y., Frank E., Wang Y., Chubinskaya S., Huang H.-H., Grodzinsky A.J. (2013). Moderate dynamic compression inhibits pro-catabolic response of cartilage to mechancial injury, TNF-alpha and IL-6, but accentuates degradation above a strain threshold. Osteoarthritis Cartilage.

[6] Ritchie M.E., Phipson B., Wu D., Hu Y., Law C.W., Shi W. (2015). Limma powers differential expression analyses for RNA-sequencing and microarray studies. Nucleic Acids Res..

[7] Altschul S.F., Madden T.L., Schäffer A.A., Zhang J., Zhang Z., Miller W. (1997). Gapped BLAST and PSI-BLAST: a new generation of protein database search programs. Nucleic Acids Res..

[8] The Gene Ontology Consortium (2000). Gene Ontology: tool for the unification of biology. Nat. Genet..

[9] The Gene Ontology Consortium (2019). The gene Ontology resource: 20 years and still GOing strong. Nucleic Acids Res..

[10] Szklarczyk D., Gable A.L., Lyon D., Junge A., Wyder S., Huerta-Cepas J. (2019). STRING v11: protein-protein association networks with increased coverage, supporting functional discovery in genome-wide experimental datasets. Nucleic Acids Res..

[11] Bateman A. (2019). UniProt: a worldwide hub of protein knowledge. Nucleic Acids Res..

[12] Good P.I. (2001).

[13] Farndale R.W., Buttle D.J., Barrett A.J. (1986). Improved quantitation and discrimination of sulphated glycosaminoglycans by use of dimethylmethylene blue. Biochim. Biophys. Acta.

[14] Struglics A., Larsson S., Pratta M.A., Kumar S., Lark M.W., Lohmander L.S. (2006). Human osteoarthritis synovial fluid and joint cartilage contain both aggrecanase- and matrix metalloproteinase-generated aggrecan fragments. Osteoarthritis Cartilage.

[15] Struglics A., Larsson S. (2010). A comparison of different purification methods of aggrecan fragments from human articular cartilage and synovial fluid. Matrix Biol..

[16] Pratta M.A., Su J.L., Leesnitzer M.A., Struglics A., Larsson S., Lohmander L.S. (2006). Development and characterization of a highly specific and sensitive sandwich ELISA for detection of aggrecanase-generated aggrecan fragments. Osteoarthritis Cartilage.

[17] Struglics A., Hansson M., Lohmander L.S. (2011). Human aggrecanase generated synovial fluid fragment levels are elevated directly after knee injuries due to proteolysis both in the inter globular and chondroitin sulfate domains. Osteoarthritis Cartilage.

[18] Laemmli U.K. (1970). Cleavage of structural proteins during the assembly of the head of bacteriophage T4. Nature.

[19] Hedbom E., Antonsson P., Hjerpe A., Aeschlimann D., Paulsson M., Rosa-Pimentel E. (1992). Cartilage matrix proteins. An acidic oligomeric protein (COMP) detected only in cartilage. J. Biol. Chem..

[20] Perez-Riverol Y., Csordas A., Bai J., Bernal-Llinares M., Hewapathirana S., Kundu D.J. (2019). The PRIDE database and related tools and resources in 2019: improving support for quantification data. Nucleic Acids Res..

[21] Swärd P., Wang Y., Hansson M., Lohmander L.S., Grodzinsky A.J., Struglics A. (2017). Coculture of bovine cartilage with synovium and fibrous joint capsule increases aggrecanase and matrix metalloproteinase activity. Arthritis Res. Ther..

[22] Said Ahmed M.A., Saweeres E.S., Abdelkader N.A., Abdelmajeed S.F., Fares A.R. (2019). Improved pain and function in knee osteoarthritis with dexamethasone phonophoresis: a randomized controlled trial. Indian J. Orthop..

[23] Hajialilo M., Ghorbanihaghjo A., Valaee L., Kolahi S., Rashtchizadeh N., Amirkhiz M.B. (2016). A double-blind randomized comparative study of triamcinolone hexacetonide and dexamethasone intra-articular injection for the treatment of knee joint arthritis in rheumatoid arthritis. Clin. Rheumatol..

[24] Duke University (2014). https://clinicaltrials.gov/show/nct02271698.

[25] Krishnan Y., Rees H.A., Rossitto C.P., Kim S.-E., Hung H.-H.K., Frank E.H. (2018). Green fluorescent proteins engineered for cartilage-targeted drug delivery: insights for transport into highly charged avascular tissues. Biomaterials.

[26] Bajpayee A.G., De la Vega R.E., Scheu M., Varady N.H., Yannatos I.A., Brown L.A. (2017). Sustained intra-cartilage delivery of low dose dexamethasone using a cationic carrier for treatment of post traumatic osteoarthritis. Eur. Cell. Mater..

[27] Evans C.H., Kraus V.B., Setton L.A. (2014). Progress in intra-articular therapy. Nat. Rev. Rheumatol..

[28] McAlindon T.E., LaValley M.P., Harvey W.F., Price L.L., Driban J.B., Zhang M. (2017). Effect of intra-articular triamcinolone vs saline on knee cartilage volume and pain in patients with knee osteoarthritis a randomized clinical trial. JAMA, J. Am. Med. Assoc..

[29] Anderson D.D., Chubinskaya S., Guilak F., Martin J.A., Oegema T.R., Olson S.A. (2011). Post-traumatic osteoarthritis: improved understanding and opportunities for early intervention. J. Orthop. Res..

[30] Wang Y., Grodzinsky A.J. (2015). The response of cartilage to injury. Post-Traumatic Arthritis Pathog. Diagnosis Manag..

[31] Carbone A., Rodeo S. (2017). Review of current understanding of post-traumatic osteoarthritis resulting from sports injuries. J. Orthop. Res..

[32] Rose B.J., Kooyman D.L. (2016). A tale of two joints: the role of matrix metalloproteases in cartilage biology. Dis. Markers.

[33] Kadler K.E., Hill A., Canty-Laird E.G. (2008). Collagen fibrillogenesis: fibronectin, integrins, and minor collagens as organizers and nucleators. Curr. Opin. Cell Biol..

[34] Eyre D.R., Weis M.A., Wu J.J. (2008). Advances in collagen cross-link analysis. Methods.

[35] Farjanel J., Sève S., Borel A., Sommer P., Hulmes J.S. (2005). Inhibition of lysyl oxidase activity can delay phenotypic modulation of chondrocytes in two-dimensional culture. Osteoarthritis Cartilage.

[36] Sanchez C., Bay-Jensen A.C., Pap T., Dvir-Ginzberg M., Quasnichka H., Barrett-Jolley R. (2017). Chondrocyte secretome: a source of novel insights and exploratory biomarkers of osteoarthritis. Osteoarthritis Cartilage.

[37] Nakayama N., Han C.Y.E., Cam L., Lee J.I., Pretorius J., Fisher S. (2004). A novel chordin-like BMP inhibitor, CHL2, expressed preferentially in chondrocytes of developing cartilage and osteoarthritic joint cartilage. Development.

[38] Aki T., Hashimoto K., Ogasawara M., Itoi E. (2018). A whole-genome transcriptome analysis of articular chondrocytes in secondary osteoarthritis of the hip. PloS One.

[39] Zhang Q., Yin Z.S., Zhang F.W., Cao K., Sun H.Y. (2018). CTHRC1 mediates il-1β-induced apoptosis in chondrocytes via JNK1/2 signaling. Int. J. Mol. Med..

[40] Durmus T., LeClair R.J., Park K.S., Terzic A., Yoon J.K., Lindner V. (2006). Expression analysis of the novel gene collagen triple helix repeat containing-1 (Cthrc1). Gene Expr. Patterns.

[41] Baud’huin M., Duplomb L., Teletchea S., Ruiz-Velasco C., Maillasson M., Redini F. (2013). Osteoprotegerin: multiple partners for multiple functions. Cytokine Growth Factor Rev..

[42] Simonet W.S., Lacey D.L., Dunstan C.R., Kelley M., Chang M.S., Lüthy R. (1997). Osteoprotegerin: a novel secreted protein involved in the regulation of bone density. Cell.

[43] Blanco F.J., Ruiz-Romero C. (2013). New targets for disease modifying osteoarthritis drugs: chondrogenesis and Runx1. Ann. Rheum. Dis..

[44] Taylor D.K., Meganck J.A., Terkhorn S., Rajani R., Naik A., O'Keefe R.J. (2009). Thrombospondin-2 influences the proportion of cartilage and bone during fracture healing. J. Bone Miner. Res..

[45] Poole C.A., Ayad S., Schofield J.R. (1988). Chondrons from articular cartilage: I. Immunolocalization of type VI collagen in the pericellular capsule of isolated canine tibial chondrons. J. Cell Sci..

[46] Tang X., Muhammad H., McLean C., Miotla-Zarebska J., Fleming J., Didangelos A. (2018). Connective tissue growth factor contributes to joint homeostasis and osteoarthritis severity by controlling the matrix sequestration and activation of latent TGFβ. Ann. Rheum. Dis..

[47] Okada H., Kikuta T., Inoue T., Kanno Y., Ban S., Sugaya T. (2006). Dexamethasone induces connective tissue growth factor expression in renal tubular epithelial cells in a mouse strain-specific manner. Am. J. Pathol..

[48] Hausser H.J., Decking R., Brenner R.E. (2004). Testican-1, an inhibitor of pro-MMP-2 activation, is expressed in cartilage. Osteoarthritis Cartilage.

[49] Grimstein C., Choi Y.K., Wasserfall C.H., Satoh M., Atkinson M.A., Brantly M.L. (2011). Alpha-1 antitrypsin protein and gene therapies decrease autoimmunity and delay arthritis development in mouse model. J. Transl. Med..

[50] Dwivedi G., Flaman L., Frank E., Geishecker E., Rosen V., Chubinskaya S. (2019). Human cartilage-bone-synovium microphysiological system to study ptoa pathogenesis and treatment on earth and in space. Osteoarthritis Cartilage.

