# Supplementary material for: Proteomic analysis reveals dexamethasone rescues matrix breakdown but not anabolic dysregulation in a cartilage injury model
Source: Osteoarthr Cartil Open. 2020 Sep 5;2(4):100099. doi: 10.1016/j.ocarto.2020.100099 (PMC8315049; doi:10.1016/j.ocarto.2020.100099)
Supplement: Multimedia component 3 [file mmc3.docx]

**SUPPLEMENTAL METHODS**

**Culture medium preparation for mass spectrometry**

Culture medium (50µL) from five different time points (days 4, 8, 12, 16 and 20, **Figure 1**) was first reduced by 4 mM dithiothreitol for 30 minutes at 56°C, alkylated by 16 mM iodoacetamide for 60 min in the dark at room temperature, and digested by 0.25 µg trypsin gold (Promega) in 0.1 M ammonium bicarbonate (AMBIC) pH 7.8 for 16 hours on a shaker at 37°C. After drying, samples were re-suspended in 100 µl 0.5 M AMBIC, run through 30 kDa filter (PALL Life Sciences) and desalted with reversed-phase C18 columns (SEM SS04V-SS18V, Harvard Apparatus) according to the manufacturer’s instructions.

**Discovery proteomics**

Discovery experiments using non-targeted mass spectrometry (MS) were performed on processed explant medium samples using a quadrupole Orbitrap benchtop mass spectrometer (Q-Exactive, Thermo Scientific) equipped with a nano-ESI interface (New Objective). A spray voltage of +2000 V was used with a heated ion transfer setting of 275°C for desolvation. The on-line reverse-phase separation was performed on an Easy nano-LC 1000 system (Thermo Scientific) using a flow rate of 300 nL/min and a linear binary gradient from 3% solvent B (acetonitrile with 0.1% formic acid; solvent A = 0.1% formic acid) for 60 min to 35% B, then to 90% for 5 min and finally isocratic 90% B for 5 min. Separation was performed on 75 μm × 25 cm capillary columns (Acclaim Pepmap RSLC, C18, 2μm, 100Å, Thermo Scientific). An MS scan (400–1200 m/z) was recorded in the Orbitrap mass analyzer set at a resolution of 70,000 at 200 m/z, 1×10^6^ automatic gain control target and 100 ms maximum ion injection time. The MS was followed by data-dependent high energy collision-induced dissociation (HCD) MS/MS scans at a resolution of 15,000 on the 15 most intense multiply charged ions at 2 × 10^4^ intensity threshold, and dynamic exclusion enabled for 30 seconds.

**Proteomics database search**

Identification from discovery data was performed using the Bos Taurus taxonomy (23969 sequences) setting of the UniProt database (UP_000009136 from 2017-10) with Proteome Discoverer 2.2 (Thermo Scientific). The processing workflow consisted of the following nodes: Spectrum Selector for spectra pre-processing (precursor mass range: 350–5000 Da; S/N Threshold: 1.5), Sequest-HT search engine (Enzyme: Trypsin; Max. missed cleavage sites: 2; peptide length range 6–144 amino acids; precursor mass tolerance: 10 ppm; fragment mass tolerance: 0.02 Da; static modification: cysteine carbamidomethylation; dynamic modification: methionine oxidation, hydroxyproline and pyro-glutamic acid (N-terminal Glu to pyroglutamic acid), and Percolator for peptide validation (false discovery rate (FDR) <1% based on peptide *q*-value). Results were filtered to keep only the Master protein with at least one unique peptide, and protein grouping was allowed according to the parsimony principle. The protein FDR was set to 0.01. Multiple peptides were measured for each protein using discovery proteomics, label-free quantification was obtained by summing up peak area intensities from unique peptides for each protein. Peptide intensities were quantified using an algorithm with feature detection and matching in Proteome Discoverer 2.2. The mass spectrometry proteomics data have been deposited to the ProteomeXchange Consortium via the PRIDE partner repository[20] with the dataset identifier PXD020756.

**Western blots of aggrecan and cartilage oligomeric matrix protein (COMP) fragments**

To analyze the proteolytic breakdown of aggrecan, samples released to the medium from one animal on day 2, 4, 8, 12, 16, 20 and 22 were deglycosylated with chondroitinase ABC (EC 4.2.2.4, Sigma), keratanase (EC 3.2.1.103, Seikagaku) and keratanase II (*Bacillus* sp. *Ks36*, Seikagaku) as previously described[14], with the exception that keratanase II incubation was done for 3h with 0.01mU/µg sGAG. Deglycosylated samples were precipitated with ice-cold acetone, and proteins were collected by centrifugation, dissolved in 2x-concentrated sample-buffer (NuPAGE, Novex, Thermo Fisher) and separated on 3-8% Tris-acetate mini-gels. After transfer to polyvinylidene difluoride (PVDF) membranes, immunoreactions were performed as described[15] using anti-ARGS (neoepitope monoclonal OA-1 antibody[16], 7 µg/ml,) or anti-G3 (polyclonal antibody, 5 µg/ml, Pierce PA1-1745) primary antibodies together with secondary peroxidase conjugated antibodies of horse anti-mouse IgG (1:25 000, Cell Signaling Technology #7076) and goat anti-rabbit IgG (20 ng/ml KPL #074-1516), respectively. The immunobands were visualized using ECL 2 Western Blotting substrate (Pierce #80196) together with luminescence image analyzer (ChemiDoc MP, BioRad) and film (Hyperfilm ECL, Amersham).

To analyze the breakdown of COMP, twenty microliters of culture media taken every second day from a separate set of bovine explant cultures (control (A) and injury plus cytokines (D)) were mixed with 2X concentrated sodium dodecyl sulfate-polyacrylamide gel electrophoresis (SDS-PAGE) sample buffer[17] without reducing agent and separated on 4-16% gradient SDS-polyacrylamide gels. After electrophoresis, proteins were transferred onto a nylon membrane (Fluorotrans transfer membrane, PVM 020C 3R, NH 1547, 0.2 μm, PALL Life Sciences) at 100 V for 2 h in 25 mM Tris, 192 mM Glycine, 10% methanol. Membranes were blocked with 3% bovine serum albumin in TBS-T (20 mM Tris-HCl, 150 mM NaCl, 0.1 % Tween-20) overnight at 4°C and incubated with a rabbit anti-bovine COMP polyclonal antiserum[18] diluted 1:1000 in 3% BSA in TBS-T, for 1 hour at room temperature. After washing with TBS-T the membranes were incubated with a secondary antibody (swine anti-rabbit HRP, P0217, DAKO, Denmark) diluted 1:5000 in 3% BSA-TBS-T for 1h. Following a washing step, reactive bands were visualized by incubating the membranes in the presence of a chemiluminescent HRP substrate and then exposed to CRONEX™ film (Sterling Diagnostic Imaging).
