# Supplementary figures and images for: Proteomic analysis reveals dexamethasone rescues matrix breakdown but not anabolic dysregulation in a cartilage injury model
Source: Osteoarthr Cartil Open. 2020 Sep 5;2(4):100099. doi: 10.1016/j.ocarto.2020.100099 (PMC8315049; doi:10.1016/j.ocarto.2020.100099)

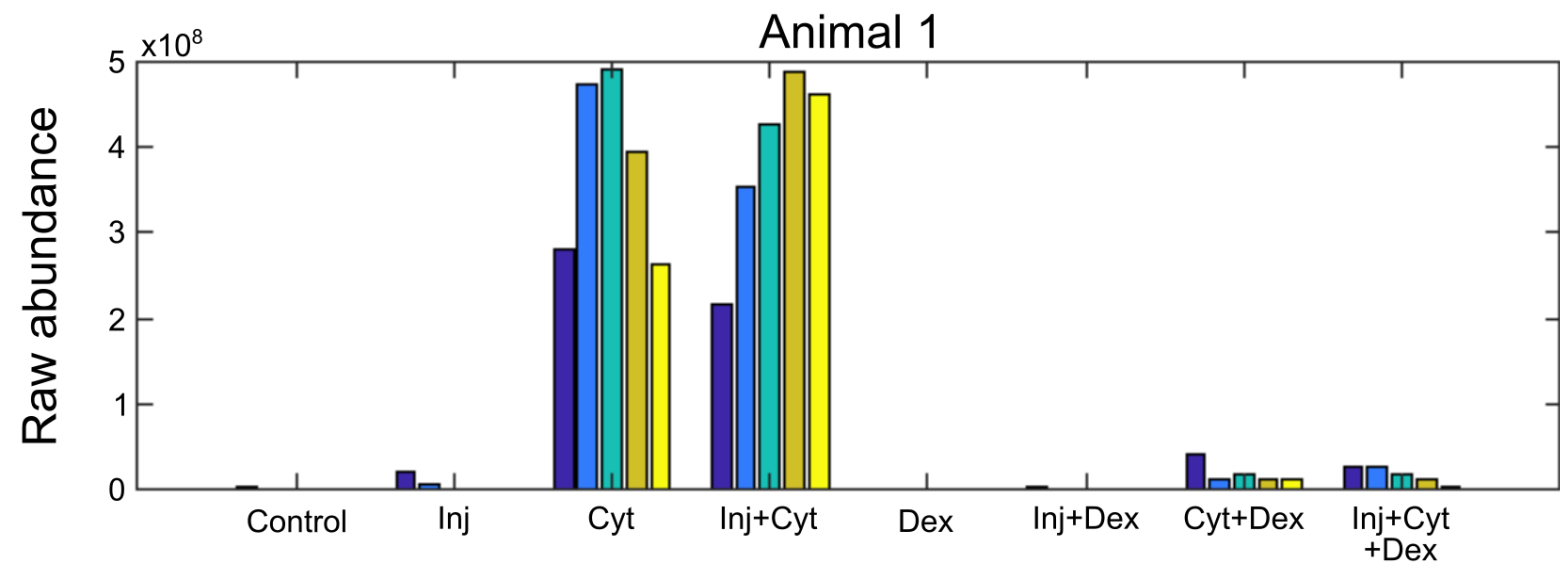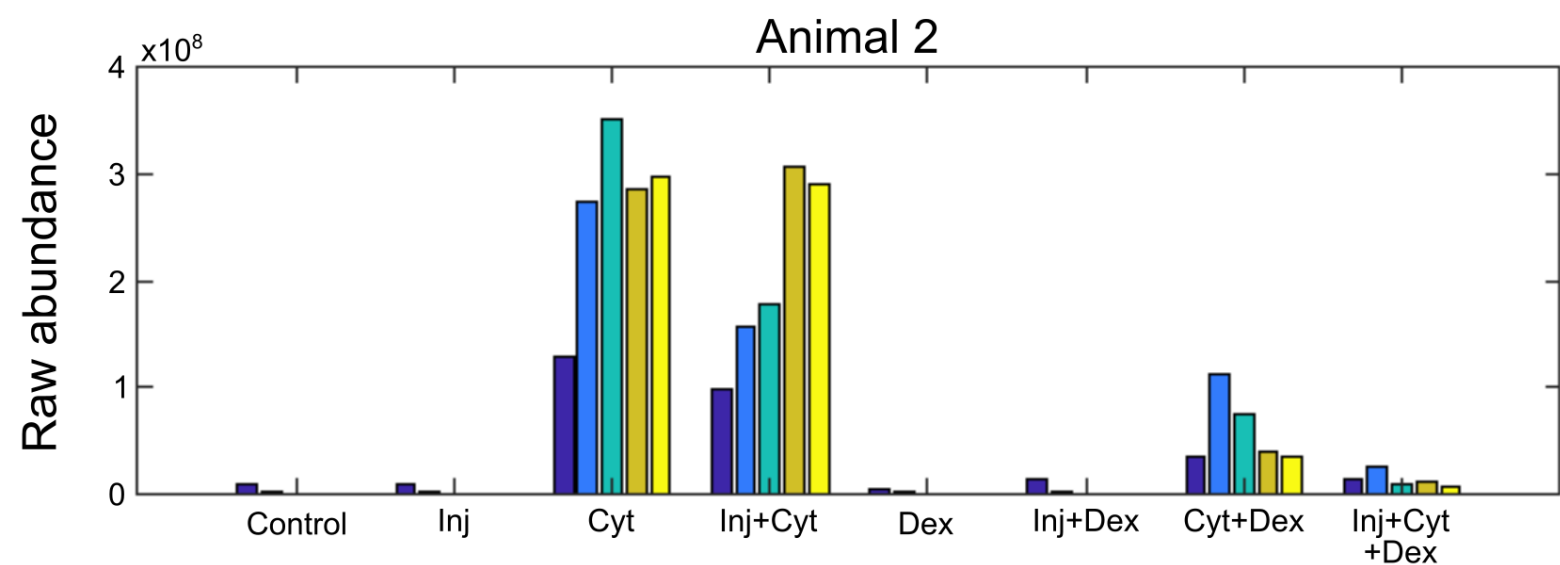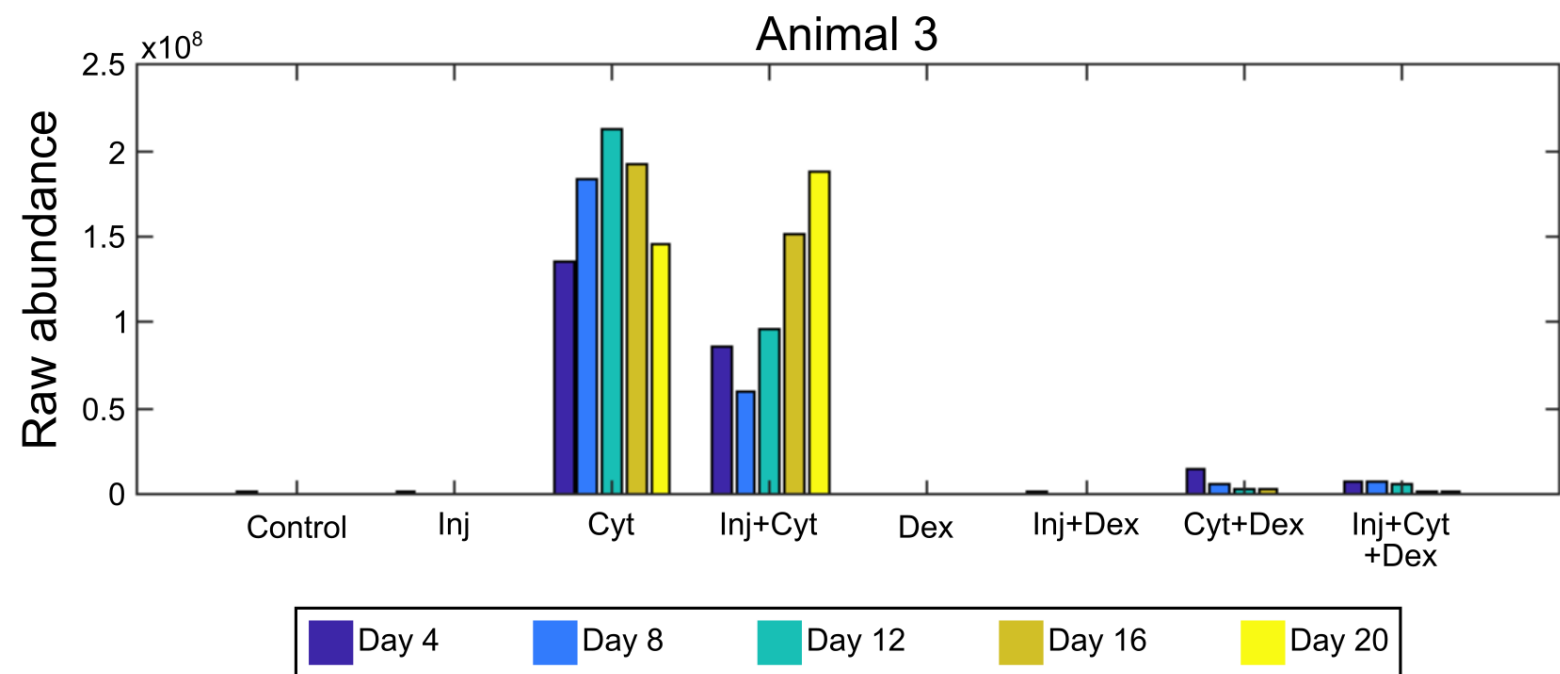

Supplement: Supplemental Fig. 1 [file mmc6.pdf]

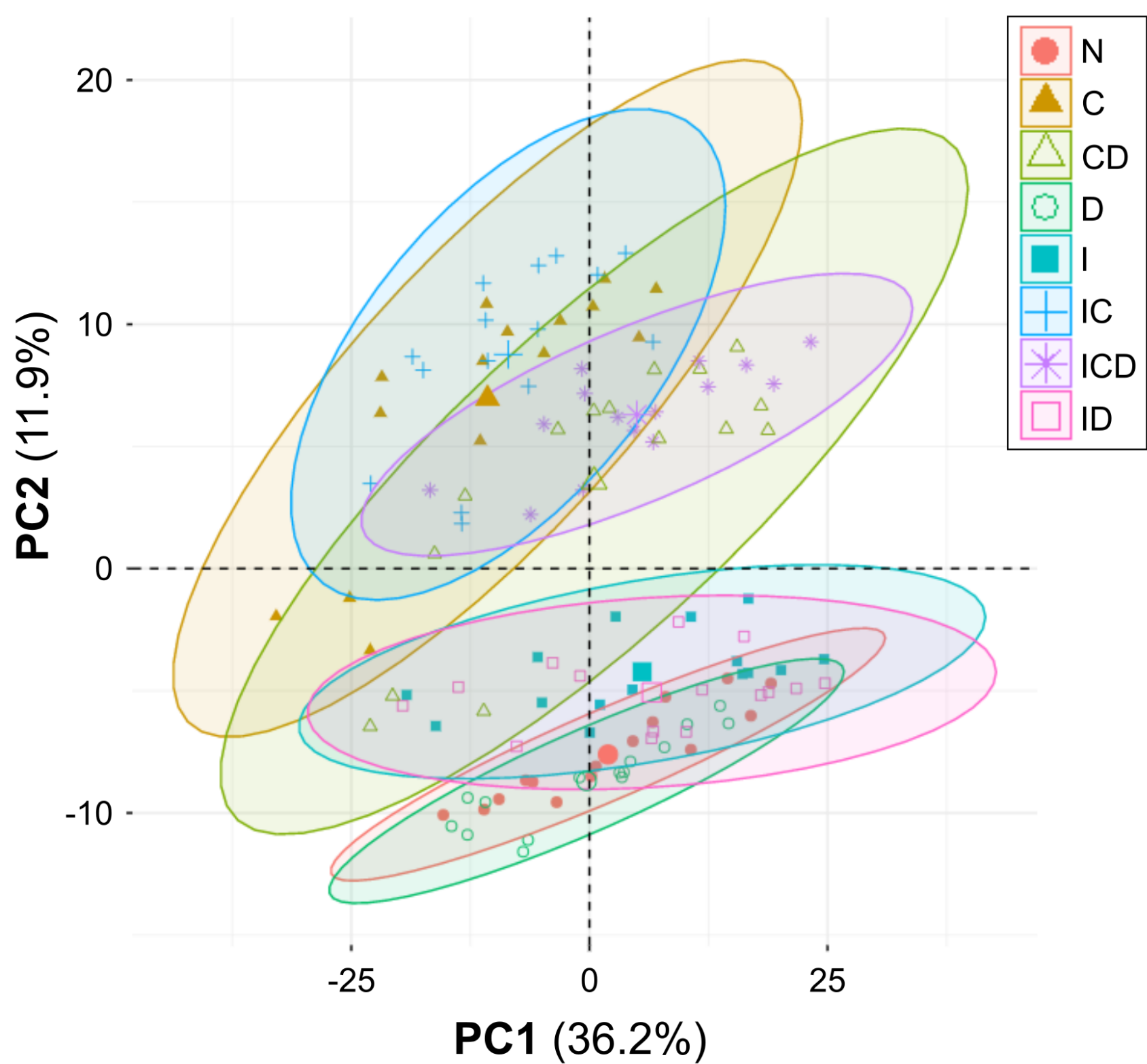

Supplement: Supplemental Fig. 2 [file mmc7.pdf]

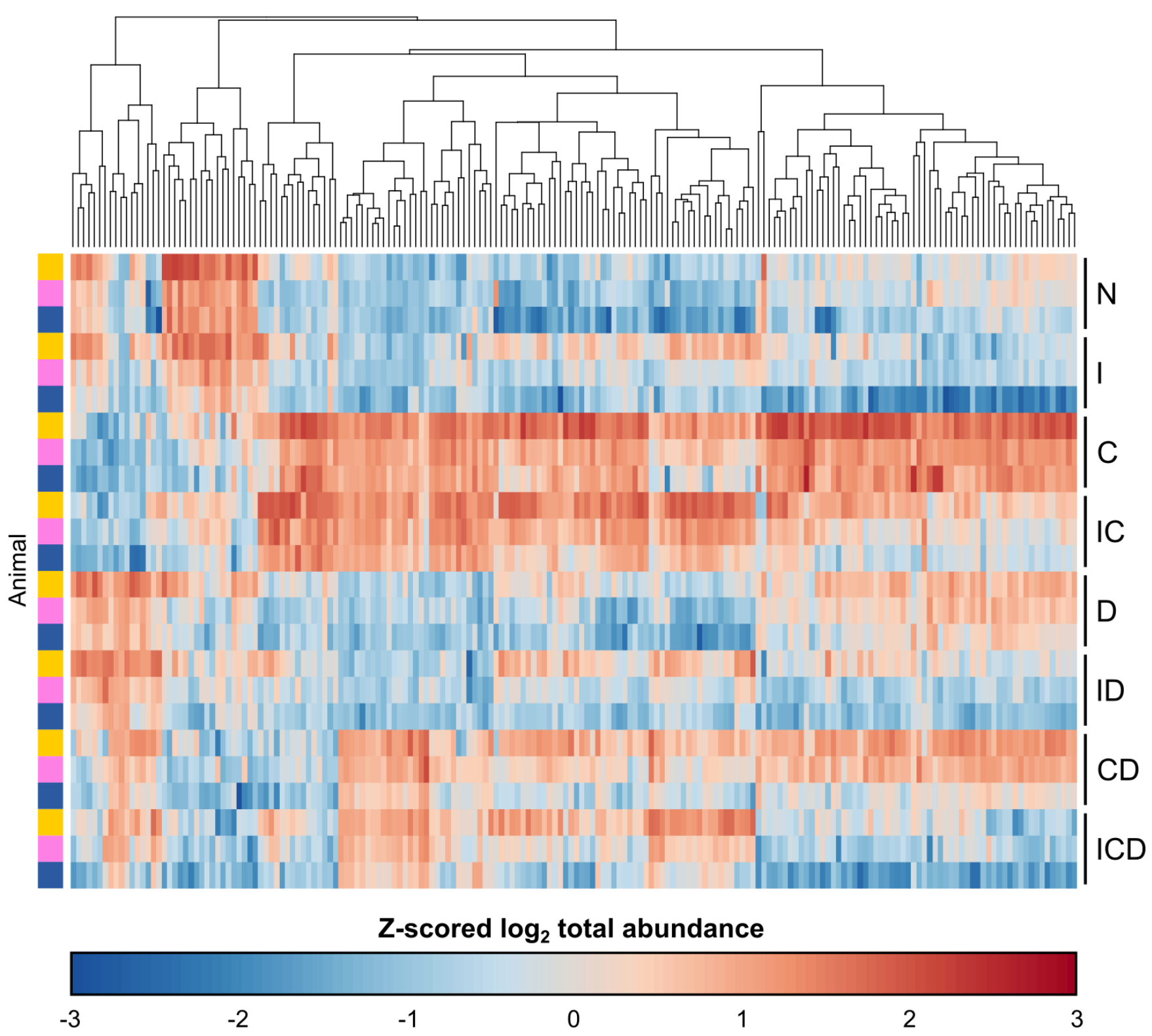

Supplement: Supplemental Fig. 3 [file mmc8.pdf]

Control

4 8 12 16 20 22

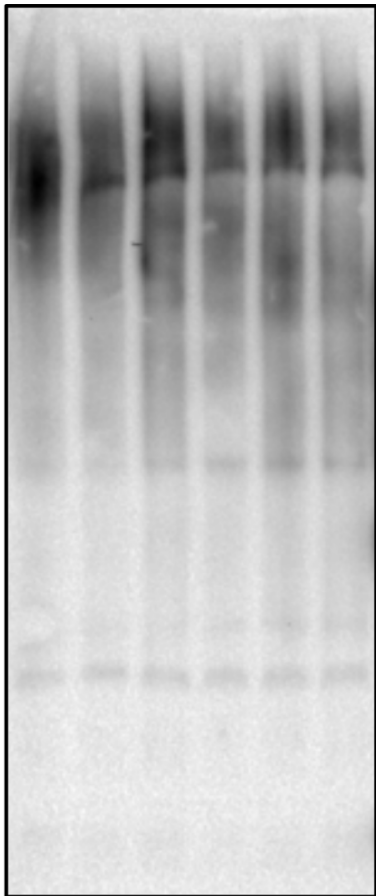

Cyt

2 4 8 12 16 20 22

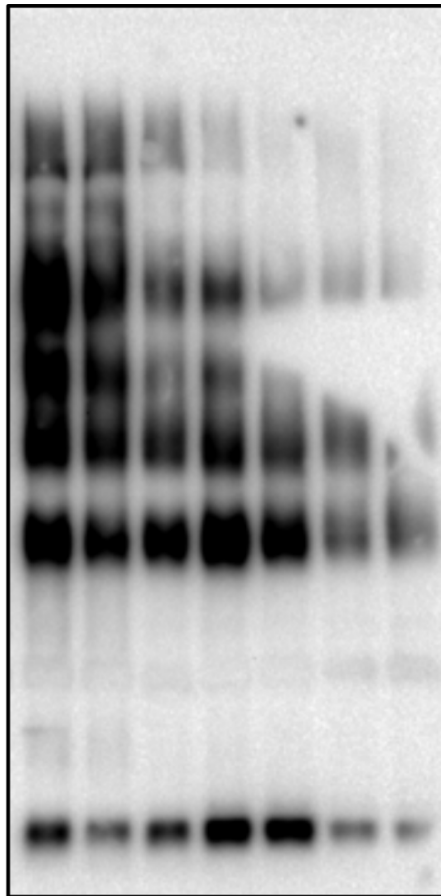

Inj+Cyt

2 4 8 12 16 20 22

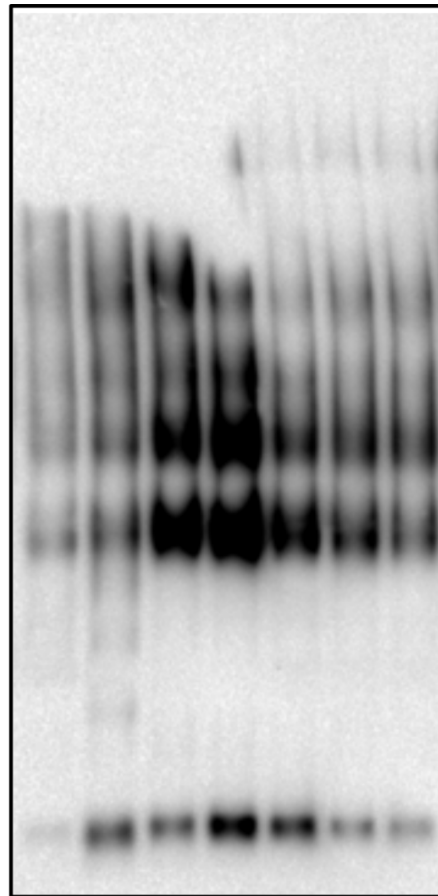

Inj+Cyt+Dex

4 8 12 16 20 22 days

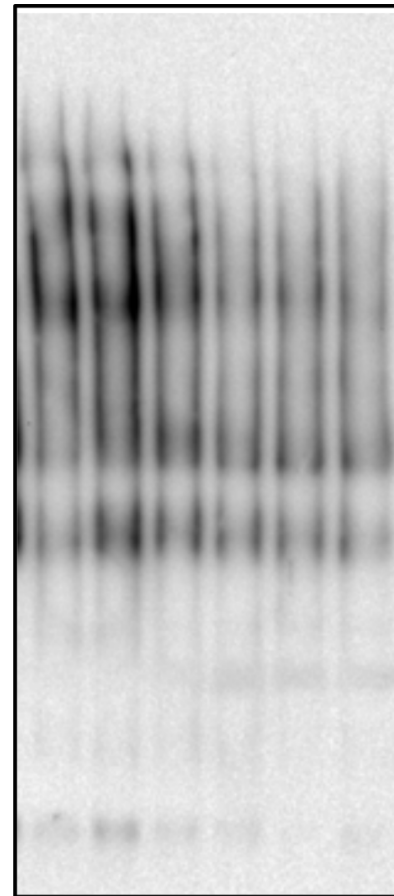

← G1-G3 (>400)

← GRGT-G3 (228)

← GLGS-G3 (182)

← AGE-G3 (139)

← LGQR-G3 (105)

← ARLE-G3 (38)

Supplement: Supplemental Fig. 4 [file mmc9.pdf]

## Control

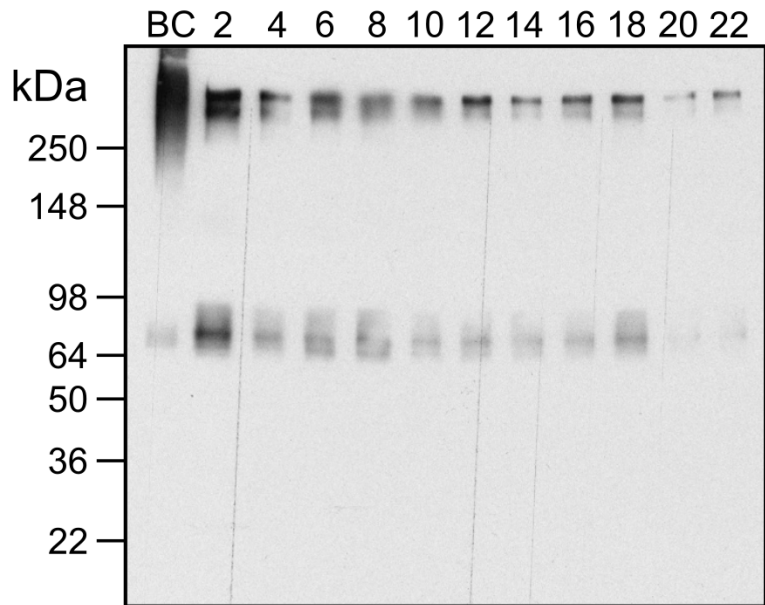

## Inj+Cyt

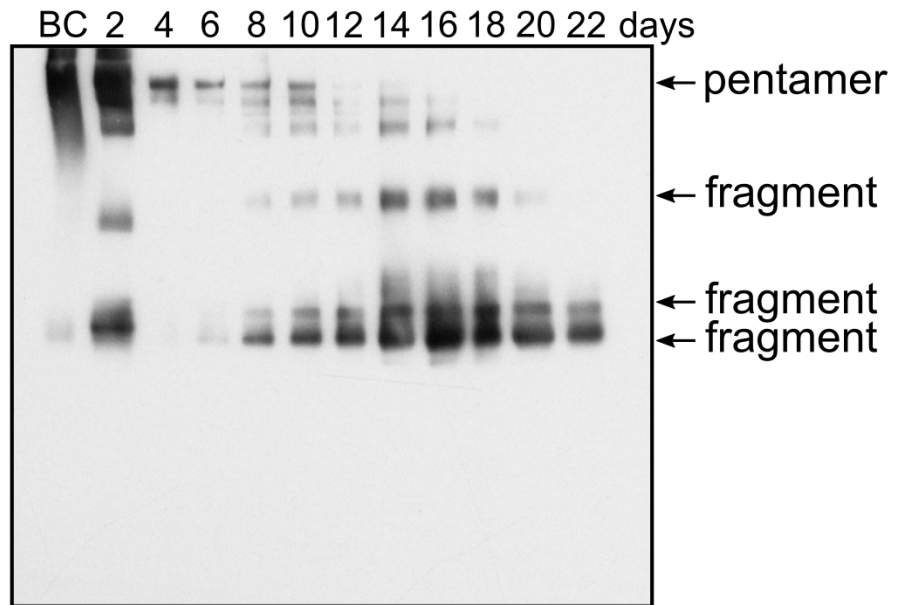

## Inj+Cyt+10% load

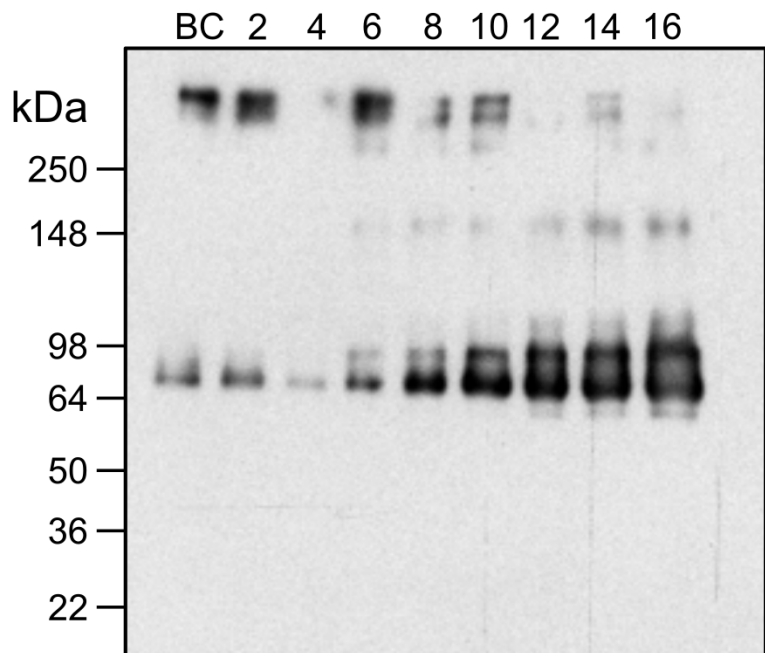

## Inj+Cyt+10% load+Dex

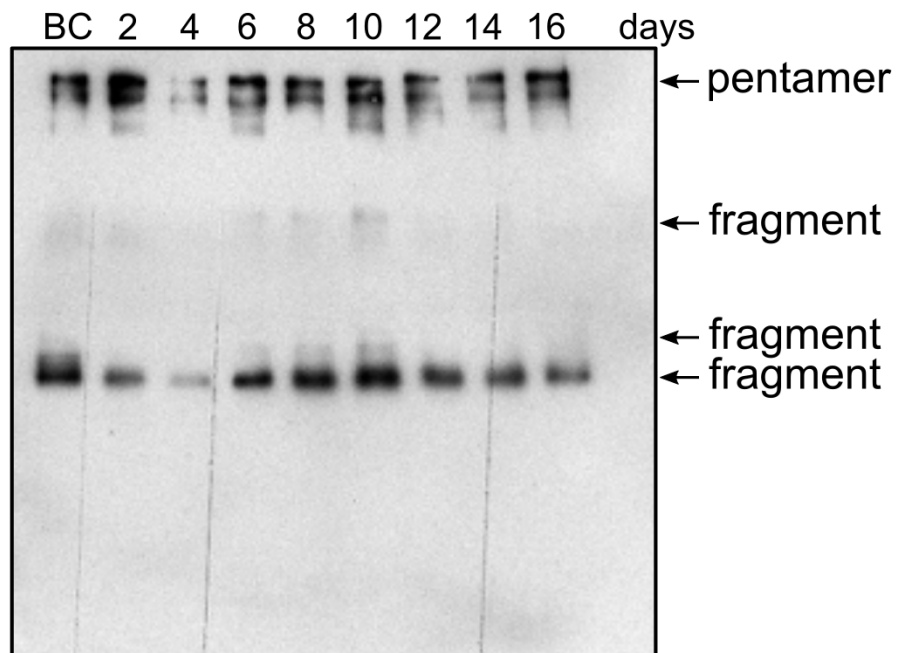

Supplement: Supplemental Fig. 5 [file mmc10.pdf]
