# Supplementary material for: Proteomic analysis reveals dexamethasone rescues matrix breakdown but not anabolic dysregulation in a cartilage injury model
Source: Osteoarthr Cartil Open. 2020 Sep 5;2(4):100099. doi: 10.1016/j.ocarto.2020.100099 (PMC8315049; doi:10.1016/j.ocarto.2020.100099)
Supplement: Multimedia component 11 [file mmc11.docx]

**Supplemental Figure S1.** Quantified protein abundance for matrix metalloprotease-1 (MMP-1), data from **Table S1**. Y-axes: area sum of uniquely identified peptides representing the protein. The colored columns from left to right show the signal/abundance at time-points 4, 8, 12, 16 and 20 days. Inj = applied injury, Cyt = addition of cytokines.

**Supplemental Figure S2. PCA for all samples.** Principle component analysis (PCA) was performed using abundance values for 456 filtered proteins obtained from MS analysis of cartilage explant medium samples taken on day 4, 8, 12, 16, and 20 of culture for all eight treatment groups: control (N), cytokines (C), cytokines+Dex (CD), Dex alone (D), injury (I), injury+cytokines (IC), injury+cytokines+Dex (ICD), and injury+Dex (ID). The cytokine-treated samples separate on PC2, and CD and ICD samples separate from their non-Dex-treated counterparts. However, Dex alone does not separate from non-treated samples, and injury+Dex broadly overlaps with injury alone. Percentages on axes represent percent variance explained by that principal component. The large symbol within each cluster represents the cluster centroid.

**Supplemental Figure S3. Heatmap of data from all treatment groups for proteins significantly affected by disease treatment.** Treatment effects were evaluated and plotted as described in Figure 3: briefly, the abundance values for the 188 selected proteins were summed, log_2_-transformed, then z-scored and clustered based on hierarchical clustering across all eight treatment conditions: control (N), injury alone (I), cytokine (C), injury+cytokines (IC), Dex (D), injury+Dex (ID), cytokines+Dex (CD), and injury+cytokines+Dex (ICD). Proteins are plotted on the horizontal axis, and ordered based on their hierarchical clustering. Each individual replicate is plotted on the vertical axis, ordered by treatment condition and then by animal.

**Supplemental Figure S4.** Western blot analysis of aggrecan G3-fragment time dependent release into the explant culture medium for treatment conditions control, cytokines (Cyt), injury+cytokines (Inj+Cyt), and injury+cytokines+Dex (Inj+Cyt+Dex). Samples were deglycosylated and run (44 to 100 µl medium/lane) on 3-8% Tris-acetate SDS-gels and applied for Western blot using anti-G3-aggrecan antibody. Representative Western blots images from full-sized blotted gels are shown. Bovine protein fragments (previously described[17,50]) are shown at the right side with the molecular mass in kDa indicated. G3 = globular domain 3.

**Supplemental Figure S5.** Time dependent release into medium of cartilage oligomeric matrix protein (COMP) detected by Western blot under different treatment conditions. Medium was collected from a separate experiment using cartilage cultured under the same conditions (control and applied injury + cytokines, Inj+Cyt), with the addition of 10% cyclic load. COMP whole pentamer proteolyzed fragments are indicated.
